# Photocatalytic Activity of Polymer Nanoparticles Modulates Intracellular Calcium Dynamics and Reactive Oxygen Species in HEK-293 Cells

**DOI:** 10.3389/fbioe.2018.00114

**Published:** 2018-08-23

**Authors:** Caterina Bossio, Ilaria Abdel Aziz, Gabriele Tullii, Elena Zucchetti, Doriana Debellis, Mattia Zangoli, Francesca Di Maria, Guglielmo Lanzani, Maria Rosa Antognazza

**Affiliations:** ^1^Center for Nano Science and Technology@PoliMi, Istituto Italiano di Tecnologia, Milan, Italy; ^2^Dipartimento di Fisica, Politecnico di Milano, Milan, Italy; ^3^Electron Microscopy Facility, Istituto Italiano di Tecnologia, Genova, Italy; ^4^Institute for Organic Synthesis and Photoreactivity, CNR-ISOF, Bologna, Italy

**Keywords:** conjugated polymer, bio-organic electronics, cell optical stimulation, Ca^2+^ imaging, reactive oxygen species, light, organic semiconductor, photomodulation

## Abstract

Optical modulation of living cells activity by light-absorbing exogenous materials is gaining increasing interest, due to the possibility both to achieve high spatial and temporal resolution with a minimally invasive and reversible technique and to avoid the need of viral transfection with light-sensitive proteins. In this context, conjugated polymers represent ideal candidates for photo-transduction, due to their excellent optoelectronic and biocompatibility properties. In this work, we demonstrate that organic polymer nanoparticles, based on poly(3-hexylthiophene) conjugated polymer, establish a functional interaction with an *in vitro* cell model (Human Embryonic Kidney cells, HEK-293). They display photocatalytic activity in aqueous environment and, once internalized within the cell cytosol, efficiently generate reactive oxygen species (ROS) upon visible light excitation, without affecting cell viability. Interestingly, light-activated ROS generation deterministically triggers modulation of intracellular calcium ion flux, successfully controlled at the single cell level. In perspective, the capability of polymer NPs to produce ROS and to modulate Ca^2+^ dynamics by illumination on-demand, at non-toxic levels, may open the path to the study of biological processes with a gene-less approach and unprecedented spatio-temporal resolution, as well as to the development of new biotechnology tools for cell optical modulation.

## Introduction

By and large, cells are essentially transparent and do not directly respond to light. Visible light is, however, a vital drive for animal behavior and species development. Accordingly, natural evolution resulted in the appearance of photoreceptors cells containing light-sensitive opsin proteins that provide a broad range of photo-induced functions (Morshedian and Fain, 2017). Photoreceptors detect light and trigger a phototransduction chain that ends in a biochemical signaling. Since long ago, scientists have explored possibilities to artificially induce light sensitivity in living tissues, in order to control physiological functions by photons. This does not simply mean copying nature in order to restore lost photo-functions in damaged organs, but it opens up a wealth of new applications in research, diagnostics and therapy. Light-induced cell control has indeed a number of advantages with respect to more traditional electrical stimulation, such as space and time resolution, reduced invasiveness and selectivity. Optogenetics is one major approach (Deisseroth, 2011). Unfortunately, in spite of the important record of experimental results, it requires the viral transfer of the sample (cell or organ) with exogenous DNA, which raises important safety constraints for application in human subjects and is still considered to be a critical limitation.

For this reason, there is a number of attempts to induce light sensitivity by a gene-less approach. The direct heating of water with a near IR laser radiation (Shapiro et al., 2012), the use of organic and inorganic devices such as photocapacitors and photodetectors (Goda and Colicos, 2006; Farah et al., 2013; Antognazza et al., 2015; Martino et al., 2015; Feyen et al., 2016; Maya-Vetencourt et al., 2017), and the introduction of conducting or semiconducting nanoparticles (Colombo et al., 2016) are among reported strategies. The latter approach is quite interesting, because it allows an easy sensitization of the target tissue and, in principle, it can select specific sites within cell sub-compartments. Nano-scaled inorganic semiconductors, such as quantum dots, are widely studied for the peculiar optical properties induced by the reduced dimensionality. Indeed, they have a huge potential for biological applications, being their dimensions close to the size of biomolecules as proteins and nucleic acids. Although there are many studies on surface functionalization being carried out, the cytotoxicity of QDs still remains an issue, halting their possible biomedical applications (Medintz et al., 2005). Despite this, they offer a strongly selective and sensitive tool for biosensing (Howes et al., 2014) and fluorescence imaging (Bruni et al., 2017). The plasmonic resonance of metallic nanoparticles has been also successfully exploited to gain efficient photo-thermal processes upon NIR radiation (Yang et al., 2017), but in this case high photo-excitation densities are usually required.

Here, we focus our attention on organic semiconducting nanoparticles (NPs), based on poly(3-hexylthiophene) (P3HT). *In vitro* use of conjugated polymer NPs has been widely reported in the literature (Tuncel and Demir, 2010; Feng et al., 2013). However, the exploitation of excellent NPs biocompatibility properties has been mainly limited, so far, to imaging and drug delivery applications (Feng et al., 2010). Conversely, their possible use as functional, light-sensitive actuators was not intensively investigated (Zangoli et al., 2017). Only very recently, we demonstrated an optical modulation effect of P3HT NPs on animal model of *Hydra vulgaris*. P3HT NPs act on the animal both at a macroscopic level, by inducing a behavioral change, and from a molecular point of view, by causing enhanced expression of opsin proteins (Tortiglione et al., 2017).

The goal of the present work is to explore the photoinduced coupling mechanism between P3HT NPs and living cells. Interestingly, we demonstrate that upon illumination polymer NPs both increase the intracellular production of reactive oxygen species (ROS) and modulate Ca^2+^ dynamics. ROS, such as the superoxide anion ${\text{O}}_{2}^{-}$ and the hydrogen peroxide H_2_O_2_, are widely involved in both physiological and pathophysiological processes (Halliwell, 2015). ROS overproduction is highly detrimental since it induces non-specific damage of proteins, lipids, and DNA (Finkel and Holbrook, 2000), causing oxidative stress (Fu et al., 2014) and leading in some cases to irreversible cell damage and apoptosis (Martindale and Holbrook, 2002). Conversely, endogenously produced ROS are essential to life (Trachootham et al., 2009), being involved in different biological functions: among others, signal transduction (Kamata and Hirata, 1999), neurotransmission (Newsholme et al., 2010), blood pressure modulation (Lee and Griendling, 2008), immune system control (Glassman, 2011), and metabolism regulation (Dröge, 2002). These versatile functions of ROS are finely modulated by their amount, duration and localization, and are currently the object of intensive investigation. Alteration in physiological ROS production leads to important pathological conditions, including autoimmune, cardiovascular, and neurodegenerative diseases, so that several ROS-responsive drug delivery systems are under investigation (Saravanakumar et al., 2017). Overall, controlled release of ROS can have highly beneficial therapeutic effects (Bergamini et al., 2004). However, suitable, fully biocompatible, precise, externally modulated and minimally invasive tools are almost completely missing at the moment. Optical modulation represents an interesting route to achieve it (Wang et al., 2012; Waiskopf et al., 2016).

Not invasive ways to modulate calcium ions flow are attracting considerable attention as well, given their key role in cell metabolism and inter-cell communication processes. Several attempts have been reported to modulate Ca^2+^ by external mediators, for instance by using magnetic NPs (Huang et al., 2010; Stanley et al., 2012; Bar-Shir et al., 2014; Lee et al., 2014; Chen et al., 2015; Wheeler et al., 2016), metallic NPs (Nakatsuji et al., 2015), organic molecules and polymers (Stein et al., 2013; Lyu et al., 2016; Takano et al., 2016) and other carbon-based materials (Miyako et al., 2014; Chechetka et al., 2016).

Interestingly, there is a close interplay between ROS and Ca^2+^ dynamics (Chakraborti et al., 1999; Brookes et al., 2004; Görlach et al., 2015), whose intensive investigation may benefit from the availability of not-invasive, locally confined and reversible tools.

Here, we show that photoexcitation of P3HT NPs internalized within Human Embryonic Kidney (HEK-293) cells leads to an increase of ROS level, associated to a variation of the intracellular Ca^2+^ dynamics. In perspective, this work may pave the way to the development of new tools for local, optical modulation of the cell physiological activity.

## Materials and methods

### Cell cultures

HEK-293 cells were grown in Dulbecco's modified Eagles' medium (D-MEM, Sigma Aldrich) with 10% fetal bovine serum (FBS, Sigma Aldrich), supplemented with 2 mM glutamine (Sigma Aldrich), 100 U/ml streptomycin (Sigma Aldrich) and 100 U/ml penicillin (Sigma Aldrich). Cells were kept in T-75 culture flasks and maintained in incubator at 37°C in a humidified atmosphere with 5% CO_2_. After reaching 80–90% of confluence, cells were detached by incubation with 0.5% trypsin-0.2% EDTA (Sigma Aldrich) for 5 min and plated for experiments. To promote cell adhesion, a layer of 2 mg/ml fibronectin (from bovine plasma, Sigma Aldrich) in phosphate buffer saline (PBS, Sigma Aldrich) was deposited on the surface of the glass coverslips and incubated for 30 min. After rinsing the fibronectin with PBS, cells were plated in their culture medium and eventually treated with polymer NPs.

### Preparation and characterization of P3HT NPs dispersions

Sterile P3HT and Polystyrene (PS) NPs were prepared by the re-precipitation method working under a laminar flow hood. P3HT (regio-regular, Sigma Aldrich) was dissolved in tetrahydrofuran (THF, Sigma Aldrich) and the resulting polymer solution was added drop-wise to sterilized water under magnetic stirring. The resulting colloidal dispersion was put in a dialysis sack and subjected to dialysis against 2 l sterilized water overnight to remove the residual organic solvent. Afterwards, the water colloidal suspension was centrifuged at different rates for 10 min, from 2,000 to 8,000 rpm, separating every time the supernatant from the precipitate, to obtain a wide range of samples with different particle dimensions (from 100 to 600 nm) and different optical density (OD). The NPs used in this work have an average hydrodynamic radius of 237 ± 82 nm, a polydispersity index of 0.12. The ODs of the dispersion administered to the cell cultures are 0.05, 0.2, 0.4. NPs are dissolved in a volume of 1 ml of culture medium 24 h before measurements. Dynamic Light Scattering (DLS) measurements were performed with a Nanobrook Omni Particle Size Analyzer, with a wavelength of 659 nm in back-scattering mode. Particles were dispersed in distilled water or cell culturing medium during analysis. Measurements were taken at 25°C. Zeta potential evaluation was performed using Smoluchowski equation. The optical absorption spectra were acquired by using Perkin Elmer Lambda 1050 UV/Vis/NIR Spectrophotometer and, for NPs upload estimation, TECAN Spark 10M Plate reader. The photoemission spectra were recorded by using HORIBA Jobin Yvon NanoLogTM spectrofluorimeter.

### Scanning electron microscopy (SEM)

Cells treated with P3HT NPs and grown on an indium-tin oxide (ITO) covered coverslip were fixed with Glutaraldehyde (Sigma Aldrich) 2.5% for 20 min. Then cells were dehydrated with incubation in solutions at increasing concentration of ethanol (Sigma Aldrich). Images were acquired with the Scanning Electron Microscope MIRA3 TESCAN.

### Transmission electron microscopy (TEM)

HEK293 cells were incubated with P3HT NPs for 24 h and were processed following a published protocol (Marotta et al., 2013). Briefly the cells after incubation were fixed for 1 h in 1.2% glutaraldehyde in 0.1 M sodium cacodylate buffer (Sigma Aldrich, pH 7.4), post fixed in 1% osmium tetroxide (Sigma Aldrich) in the same buffer and en bloc stained with 1% uranyl acetate aqueous solution. The cells were then dehydrated in a graded ethanol series and embedded in epoxy resin (Epon 812, TAAB). Semi-thin and thin sections of the embedded cell monolayer were cut with an ultramicrotome (UC6, Leica) equipped with a diamond knife (Diatome). Projection images were acquired using a JEOL JEM 1011 transmission electron microscope operating at at 100 kV of acceleration voltage and recorded with a 2 Mp charge-coupled device (CCD) camera (Gatan Orius SC100).

### Immunocytochemical studies

Cells treated with P3HT NPs grown on fibronectin-coated glass coverslips were washed twice with PBS and fixed for 20 min at RT in 4% paraformaldehyde (Sigma Aldrich) and 4% sucrose (Sigma Aldrich) in 0.12 M sodium phosphate buffer, pH 7.4. Labeling with phalloidin and fluorescein Isothiocyanate (Sigma Aldrich) was applied in GDB buffer (30 mM phosphate buffer, pH 7.4, containing 0.2% gelatin, 0.5% Triton X-100 and 0.8 M NaCl (Sigma Aldrich) for 2 h at RT.

### Viability assay

In order to preliminarily evaluate the NPs cytotoxicity, the MTT [3-(4,5-dimethylthiazol-2-yl)-2,5-diphenyltetrazolium bromide] (Sigma Aldrich) assay was performed on HEK-293 cells. Cells were seeded in 12 wells plates at a density of 2 × 10^4^ cells/well, together with the NPs. Cell proliferation was evaluated after 24, 48, 72, and 96 h incubation. For each time point, the growing medium was replaced with RPMI without phenol red (Sigma Aldrich) containing 0.5 mg/ml of MTT. The samples were incubated again for 3 h at 37°C in dark. Formazan salt produced by cells through reduction of MTT was then solubilized with 200 μl of ethanol and the absorbance was read at 560 and 690 nm. The proliferation cell rate was calculated as the difference in absorbance at 560 and 690 nm. Photocytoxicity of NPs- treated cells subjected to optical excitation has been evaluated by Fluorescein Diacetate (FDA, Sigma Aldrich) assay. FDA is a non-fluorescent molecule, which is hydrolyzed to fluorescent fluorescein in viable cells only. Different cohorts of HEK-293 cells (treated/untreated with P3HT NPs, subjected/not subjected to photoexcitation) were incubated for 5 min with FDA 5 μM. After careful wash outs of the excess FDA from the extracellular medium and repeated rinses with physiological saline solution, representative images were acquired (excitation/emission wavelengths, 490/520 nm, integration time 100 ms) using an inverted fluorescence microscope (Nikon Eclipse Ti; 20× objective), equipped with an Analog-WDM Camera (Cool Snap Myo, Photometrics). Photocytotoxicity tests have been carried out in different experimental conditions, namely: (i) at different NPs doses, corresponding to OD values in the range 0–0.5, at 2 DIV/24 h after illumination. The photoexcitation protocol used in FDA assay is the same employed in ROS detection experiments (see below); (ii) at fixed NP dose, corresponding to OD = 0.2, at different time points after the photoexcitation protocol, up to 4 DIV/72 h after illumination. Specific care was taken in order to image, at different time points, exactly the same sample area previously subjected to optical excitation. All experiments were carried out in relevant control samples as well (untreated and/or not illuminated cell cultures).

### Chronoamperometry measurements

Photocurrent measurement was carried out by using a potentiostat/galvanostat (Autolab, PGSTAT 302N). The electrochemical cell was in three-electrode configuration and was divided into two compartments, connected by a saline bridge. One compartment contained the reference and the counter electrodes, a saturated KCl Ag/AgCl and Pt wire respectively, immersed in the pure electrolyte (phosphate buffer 10 mM, pH 7). The second compartment contained the Indium-Tin-Oxide (XynYan Technology, 15 nm thickness, sheet resistance 15 ohm/sq) working electrode and the P3HT NPs dissolved in the same electrolyte solution. Chronoamperometry measurements were performed at the electrochemical equilibrium, by applying a potential equal to the open circuit potential (OCP), and by changing the applied voltage in the range (OCP - 300 mV) ≤ V ≤ OCP vs. Ag/AgCl. A continuous light source (Thorlabs LED M470L3-C5, 470 nm central emission wavelength) was used for the photoexcitation, with a power density of 2.7 mW/mm^2^. The photocurrent data at fixed wavelengths were obtained by exciting the NPs dispersion with six CW LED light sources in the visible spectrum. Peak emission wavelengths and corresponding power densities are: λ_1_ = 470 nm, P_D1_ = 3.45 mW/mm^2^; λ_2_ = 505 nm, P_D2_ = 1.69 mW/mm^2^, λ_3_ = 530 nm, P_D3_ = 1.36 mW/mm^2^, λ_4_ = 617 nm, P_D4_ = 2.02 mW/mm^2^, λ_5_ = 627 nm, P_D5_ = 2.01 mW/mm^2^, λ_6_ = 655 nm, P_D6_ = 2.21 mW/mm^2^).

### Reactive oxygen species (ROS) detection

Dichlorofluorescein diacetate (H_2_DCF–DA, Sigma Aldrich), 2-[6-(4′-amino)-phenoxy-3H-xanthen-3-on-9-yl] benzoic acid (APF, Sigma Aldrich) and 2-[6-(4′-Hydroxy)-phenoxy-3H-xanthen-3-on-9-yl] benzoic acid (HPF, Sigma Aldrich) were employed for intracellular detection of Reactive Oxygen Species (ROS). Sterile P3HT and PS NPs were administered to the cells at the plating step, by diluting them in the extracellular medium up to the desired concentration, corresponding to OD = 0.05, 0.2, 0.4. At 1DIV HEK-293 cells already showed uniform coverage of the substrate (about 40% confluence) and NPs were efficiently internalized within the cell cytosol. Excess, non-internalized NPs were removed by subsequent, repeated rinses of the extracellular medium. NPs treated cells and control, untreated cells were photo-excited by illuminating each sample for 2 min with a LED system (Lumencor Spectra X light engine, λ = 540 nm, P_D_ = 95 mW/mm^2^). Subsequently, cells were incubated with the ROS probes for different times according to the employed probe (t_H2DCF−DA_ = 30 min, t_APF_ = t_HPF_ = 40 min). After careful wash-out of the excess probe from the extracellular medium, the fluorescence of the probes was recorded (excitation/emission wavelengths, 490/520 nm; integration time, 70 ms for H_2_DCF–DA and 500 ms for HPF e APF) with an inverted microscope (Nikon Eclipse Ti), equipped with an Analog-WDM Camera (Cool Snap Myo, Photometrics). The same protocol was employed for control, untreated cells. Variation of fluorescence intensity was evaluated over Regions of Interest covering single cells areas, and reported values represents the average over multiple cells. See figure captions and SI for additional details about statistical analysis. Image processing was carried out with ImageJ and subsequently analyzed with Origin 8.0.

### Intracellular Ca^2+^ measurements

HEK-293 cultures were loaded for 30 min at 37°C with 1 μM Fluo-4 (Life Technologies) in extracellular solution (5 mM HEPES; 135 mM NaCl, 5,4 mM KCl, 1 mM MgCl_2_, 1,8 mM CaCl_2_, 10 mM glucose, all purchased from Sigma Aldrich; pH 7.4) and Ca^2+^ free extracellular solution (10 mM HEPES; 150 mM NaCl, 3 mM MgCl_2_, 5 mM EGTA, all purchased from Sigma Aldrich; pH 7.4). Then, cells were washed for 10 min with pre-warmed extracellular solution before recordings. Cells treated with P3HT and PS NPs, as well as untreated samples, were illuminated for 3 min (emission peak wavelength, 485 nm; photoexcitation density, 2.1 mW/mm^2^ and 14.6 mW/mm^2^, spot size 0.9 mm^2^) by using a LED source light (Lumencor Spectra X light engine). Videos were collected with an inverted microscope Nikon Eclipse Ti equipped with an Analog-WDM Camera (Cool Snap Myo, Photometrics). Variation of fluorescence intensity was evaluated over Regions of Interest covering single cells areas, and reported values represents the average over multiple cells. See Figure captions and SI for additional details about statistical analysis. Image processing was carried out with ImageJ and subsequently analyzed with Origin 8.0. ROS inhibition was obtained by administration of N–Acetyl–Cysteine (NAC, Sigma Aldrich), dissolved in extracellular solution (with and without Ca^2+^) at a final concentration of 10 μM.

### Statistical analysis

See SI section.

## Results

### Optical and microscopy characterization of polymer P3HT nanoparticles (P3HT NPs)

**Figure 1:**
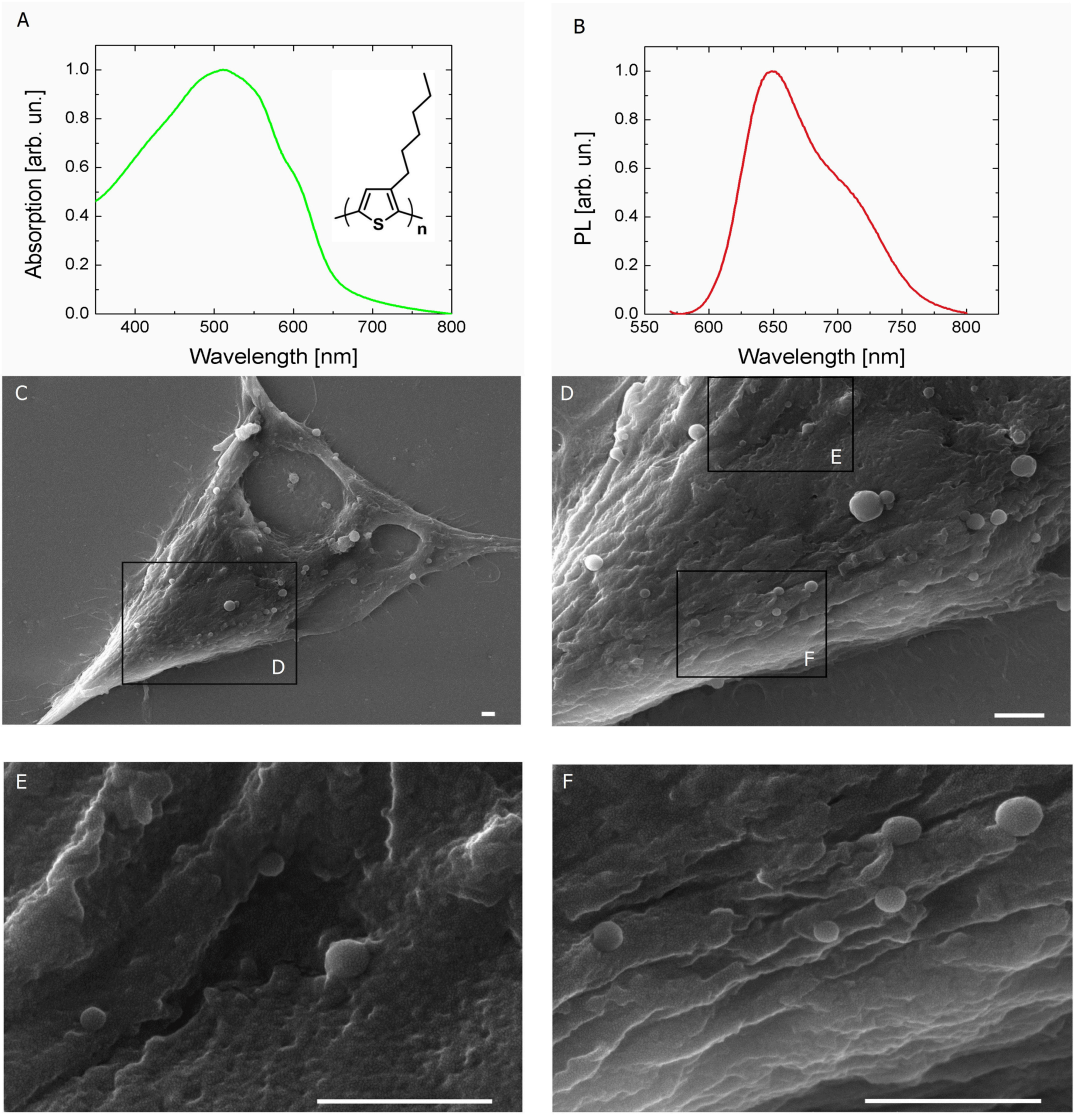
Light sensitive P3HT NPs and HEK-293 living cells. **(A,B)** Optical absorption and fluorescence (PL) spectra of P3HT NPs in aqueous dispersion. P3HT chemical structure is also shown in the inset. **(C–F)** Top view scanning electron microscopy (SEM) images of HEK-293 cells treated with P3HT NPs, at increasing magnification. Scale bar, 1 μm.

Polymer NPs, entirely constituted by poly(3-hexylthiophene), have been synthesized by the re-precipitation method in sterile conditions, as previously described (Zucchetti et al., 2016). P3HT NPs have an average hydrodynamic diameter of 237 ± 82 nm and a polydispersity index of 0.12, as measured by dynamic light scattering (Figure S1A). They also show excellent colloidal stability, being characterized by a zeta-potential value of −35 ± 8 mV. Recent literature has firmly established the importance of studying in detail the interaction between polymer-coated NPs and proteins, which largely affects cellular uptake and cytotoxicity (Hühn et al., 2013). Here, colloidal stability within the cell culturing medium (D-MEM, without the pH indicator phenol red) was evaluated over time, by carrying out both DLS measurements (i.e., by assessing modifications of the hydrodynamic diameter, Figure S1B), UV-Vis absorption and photoluminescence spectroscopy (Figures S1C,D). Three different NPs dispersions have been considered, corresponding to optical density values of 0.2, 0.4, and 0.5. In all cases, NPs dispersions do not show significant aggregation effects within the cell culturing medium, up to 48 h. The optical absorption and emission spectra of P3HT NPs aqueous dispersion are reported in Figures 1A,B, respectively (inset, P3HT chemical structure). It has been recently demonstrated that the optical properties of P3HT NPs are well preserved within the cell cytosol environment (Zucchetti et al., 2016). Preliminary studies of P3HT NPs toxicity both in Human Embryonic Kidney (HEK-293) cells (Zucchetti et al., 2016) and in *Hydra Vulgaris* animal models (Tortiglione et al., 2017) have been previously carried out in dark condition, and no clear adverse effects were reported. In this work, P3HT NPs were administered to the HEK-293 cell culturing medium at the cell plating step, and their cytotoxicity was preliminarily evaluated by measuring cellular proliferation through the MTT method. The assay relies on the capability of living cells to metabolize/reduce a water-soluble tetrazolium salt (3-(4′,5′-dimethylthiazol-2′-yl)-2,5-diphenyl-2H-tetrazolium hydrobromide, MTT) into a water-insoluble formazan product, which has a characteristic purple color. The optical absorption of formazan is thus considered a clear indicator of cell viability and capability to proliferate, being proportional to the number of living cells. Figure S2 shows the formazan optical absorption recorded in NPs -treated and -untreated cells at four different time points, 1, 2, 3, and 4 days after incubation (DIV). The presence of NPs leads in general to a reduced cell proliferation with respect to untreated samples; the absorption percentage variation between treated and untreated cell cultures is about 45% at DIV 1. This difference, however, shows a partial recovery over time, and amounts at 20%, from DIV 2 onwards, up to DIV 4. Scanning electron microscopy (SEM) images (Figures 1C–F) document the very well defined spherical geometry and the good surface smoothness of NPs. The limited presence of aggregates is a further index of the high stability properties within the cellular environment of P3HT NPs, despite the absence of any surfactant agent during the fabrication protocol. P3HT NPs can be easily visualized by fluorescence imaging, thanks to their intrinsic photoluminescence peaking in the red spectral region (Figure 1B). Effective P3HT NPs internalization within HEK-293 cell cytosol was previously assessed by confocal laser scanning microscopy (Zucchetti et al., 2016). Here, P3HT NPs cellular uptake was quantitatively estimated by using two different methods, based on fluorescence imaging (Figure S3) and variation of the optical absorption spectrum of the NPs dispersion before and after 24 h administration to the cell cultures (Figure S4). In both cases, three initial concentrations of P3HT NPs dispersions were considered, corresponding to optical density values of 0.05, 0.2, and 0.4. In the latter two cases, effective NPs upload is detected, in the order of few hundreds of NPs /cell, in agreement with previously reported CLSM data. Conversely, for the lowest considered concentration of the dispersion, a considerably lower fraction of NPs undergoes efficient internalization within the cell cytosol (see details in the SI section). Finally, for control experiments, we used light-insensitive polystyrene NPs (PS NPs). These NPs have dimensions and zeta-potential values (hydrodynamic diameter, 225 ± 72 nm; zeta-potential,−41.8 ± 1.1 mV) similar to P3HT NPs, but do not have remarkable optoelectronic properties: they have negligible absorption in the blue-green spectral range, do not generate electric charges upon optical excitation and are electrically insulating materials, thus representing a good model for control, optically and electrically inert NPs. Detailed characterization of PS NPs properties and colloidal stability within the cell culturing medium over time are reported in the SI section (Figure S5). Transmission electron microscopy (TEM) analysis corroborates SEM and fluorescence imaging showing the presence of endosome-like compartments inside the cell cytoplasm. These compartments are filled with electron-dense nanoparticles compatible in shape and size with the P3HT NPs (Figure S11).

### P3HT NPs photo-electrochemical characterization

**Figure 2:**
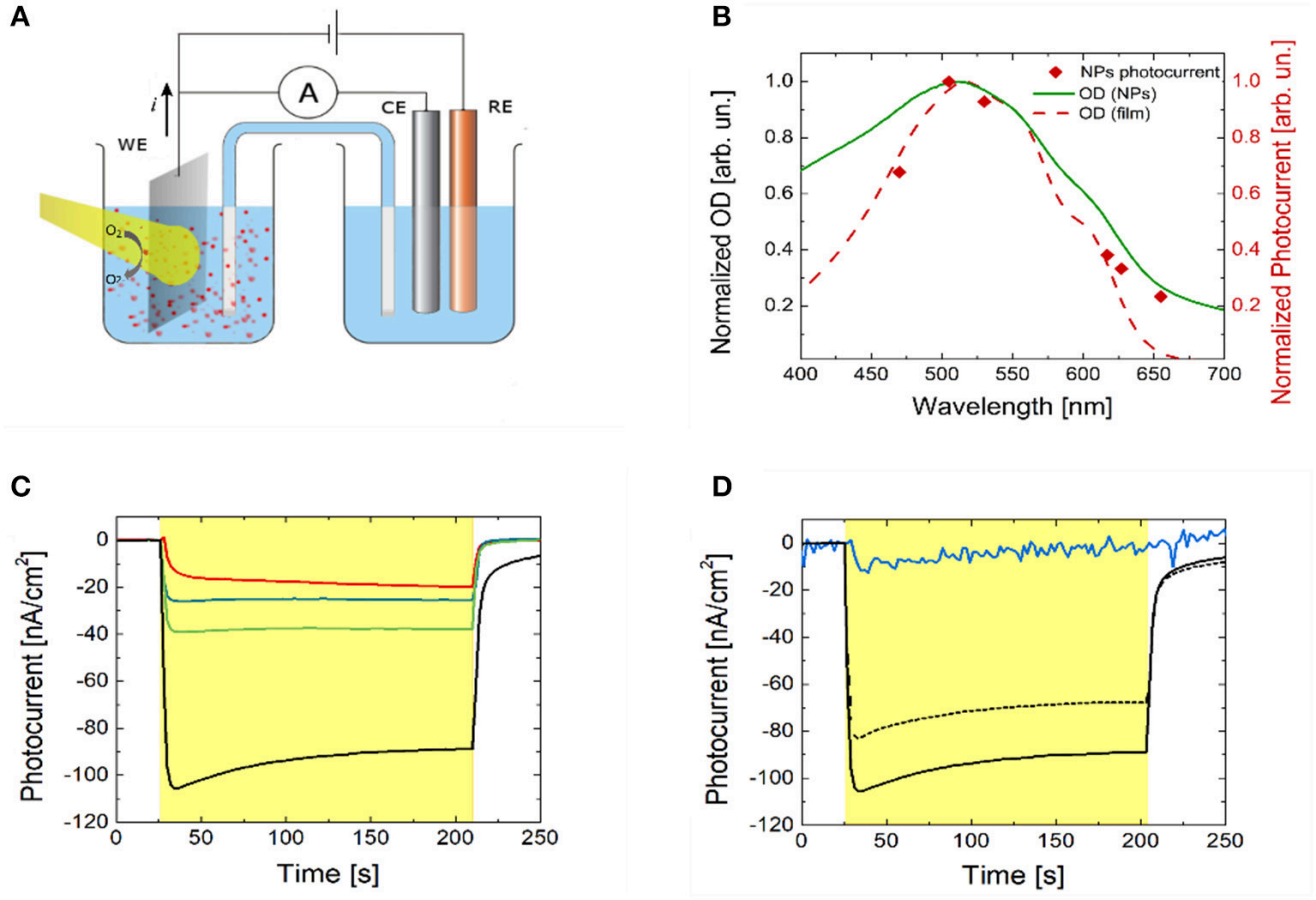
P3HT NPs photo-electrochemical characterization. **(A)** Experimental set-up used for photocurrent action spectrum and chronoamperometry experiments. A planar ITO slab, a saturated KCl Ag/AgCl, and a Pt wire are used as the back contact, the reference (RE) and the counter electrode (CE), respectively. Phosphate Buffer Saline solution at 10 mM molar concentration and pH = 7.0 has been used as the electrolyte. Light is incident from the glass side of the ITO slab. **(B)** Normalized absorption spectra of P3HT thin solid film (red dashed line) and of NPs dispersion (green solid line) are compared with the P3HT NPs photocurrent action spectrum (red diamonds). The latter was obtained by plotting the steady value of the photocurrent obtained by exciting the NPs dispersion with six narrow-band light sources covering the visible range (emission peak wavelengths at 470, 505, 530, 617, 627, 655 nm). **(C)** Photocurrent dynamics recorded in P3HT NPs dispersion (light source emission peak wavelength, 470 nm). The red line represents the photocurrent at the OCP value. The blue, green and black lines show the photocurrent signals recorded upon externally applied bias OCP–100 mV, OCP–200 mV, and OCP–300 mV, respectively. The yellow shaded area represents the temporal window corresponding to optical excitation. **(D)** Photocurrent dynamics with and without molecular oxygen in solution at V = OCP–300 mV. The black solid line represents the photocurrent recorded in ambient conditions (i.e., in equilibrium with the atmosphere). Upon removal of oxygen from the electrolyte and in sealed, nitrogen atmosphere the photocurrent signal is almost completely suppressed (blue solid line). Upon partial re-oxygenation of the electrolyte solution and recovery to the atmospheric equilibrium condition, photocurrent signal is almost completely restored to the initial value (black dashed line). The yellow shaded area represents the temporal window corresponding to optical excitation.

The photo-electrochemical activity of P3HT polymer thin films in an aqueous saline environment has been reported in a number of recent works (Lanzarini et al., 2012; Bellani et al., 2015; Giron et al., 2016; Tullii et al., 2017). The observed photocurrent generation has been attributed to photo-electrochemical reactions, leading to oxygen and, less efficiently, to hydrogen reduction. In the present case, P3HT NPs are dispersed in a phosphate buffer solution (10 mM, pH 7, optical density of 2). Their capability to photo-generate charges has been initially assessed. We carried out chrono-amperometry and spectral responsivity measurements on the NPs dispersion in a three electrode configuration, employing a planar ITO slab, a saturated KCl Ag/AgCl and a Pt wire as the working (WE), the reference (RE) and the counter electrode (CE), respectively (Figure 2A). Visible light is incident from the glass substrate of the ITO slab. In order to avoid electrode contamination by P3HT NPs, the WE, immersed in the dispersion, was separated from the RE and CE compartment by a saline bridge. Prior to chrono-amperometry measurements, the open circuit potential (OCP) value was estimated, being about 150 mV. Photocurrent has been measured at different excitation wavelengths by using six LED light sources (Figure 2B, red diamond symbols). The spectral dependence shows a symbatic behavior, closely resembling the optical absorption spectrum of the P3HT thin film (red dashed line). This indicates that the recorded current is undoubtedly due to charge photogeneration by polymer NPs and allows to exclude spurious reduction/oxidation effects. Chronoamperometry measurements have been then carried out, by applying fixed bias *V* in the range (OCP-300 mV) ≤ *V* ≤ OCP, with steps of 100 mV amplitude (Figure 2C). Upon light excitation (470 nm peak excitation wavelength, 3 min stimuli duration, 2.7 mW/mm^2^ photoexcitation density), a photocurrent signal is recorded, with increasing amplitude at increasingly negative bias. The external quantum efficiency, defined as the ratio between the number of electrons flowing in the external circuit and the number of incident photons per unit time at a fixed wavelength (470 nm in our case), is very low, in the order of 10^−6^, four orders of magnitude lower than the value obtained in hybrid solid/liquid devices based on bulk heterojunction polymer thin films, namely P3HT doped with the fullerene derivative electron acceptor phenyl-C61-butyric acid methyl ester (Lanzarini et al., 2012; Bellani et al., 2015). Still, these results unequivocally demonstrate that P3HT NPs can photo-generate free charges, which interact with the surrounding electrolyte and sustain photo-electrochemical reactions. Photocurrent dynamics recorded by using P3HT polymer samples prepared in different ways (namely, P3HT thin films spin-coated from solution onto the ITO WE, P3HT NPs thin films casted from dispersion and ITO WE previously subjected to P3HT NPs exposure) all show negative photocurrent signal, like in the case of NPs dispersion (Figure S6), thus indicating similar interfacial charge transfer mechanisms at the ITO electrode. In particular, in accordance with the observed photocurrent sign, photogenerated holes (carried by the ionized NPs or NP^+^) are efficiently transferred to the WE, while electrons react with species present in solution. In analogy with results obtained with polymer thin films (Tullii et al., 2017), we believe that the predominant photo-electrochemical reaction at NPs surface is reversible oxygen reduction, which leads to the creation of reactive oxygen species, such as ${\text{O}}_{2}^{-}$. This is fully confirmed by photocurrent measurements with P3HT NPs dispersion in de-oxygenated condition, and after partial re-oxygenation (Figure 2D).

The energetic alignment of HOMO and LUMO levels of P3HT polymer (~5 and ~3.5 eV, respectively) with respect to the ITO work function (~4.5 eV for oxygen-plasma treated substrates, well aligned with the polymer HOMO level) and to the 1-electron oxygen reduction reaction O_2_/${\text{O}}_{2}^{\text{.}-}$ (– 0.33 V vs. SHE at pH 7, corresponding to ~4.1 V in the absolute scale, well aligned with the P3HT LUMO level) are in agreement with this picture. P3HT NPs work function has been experimentally evaluated by Kelvin Probe measurements, obtaining values slightly lower than P3HT polymer thin films [4.5 vs. 4.8 eV, respectively, in line with recent literature (Di Maria et al., 2017)], which may further facilitate the occurrence of oxygen reduction reactions.

### Production of reactive oxygen species (ROS)

Photocurrent measurements demonstrate the occurrence of photo-electrochemical reactions, possibly involving reactive oxygen species (ROS) as intermediates and/or final products.

**Figure 3:**
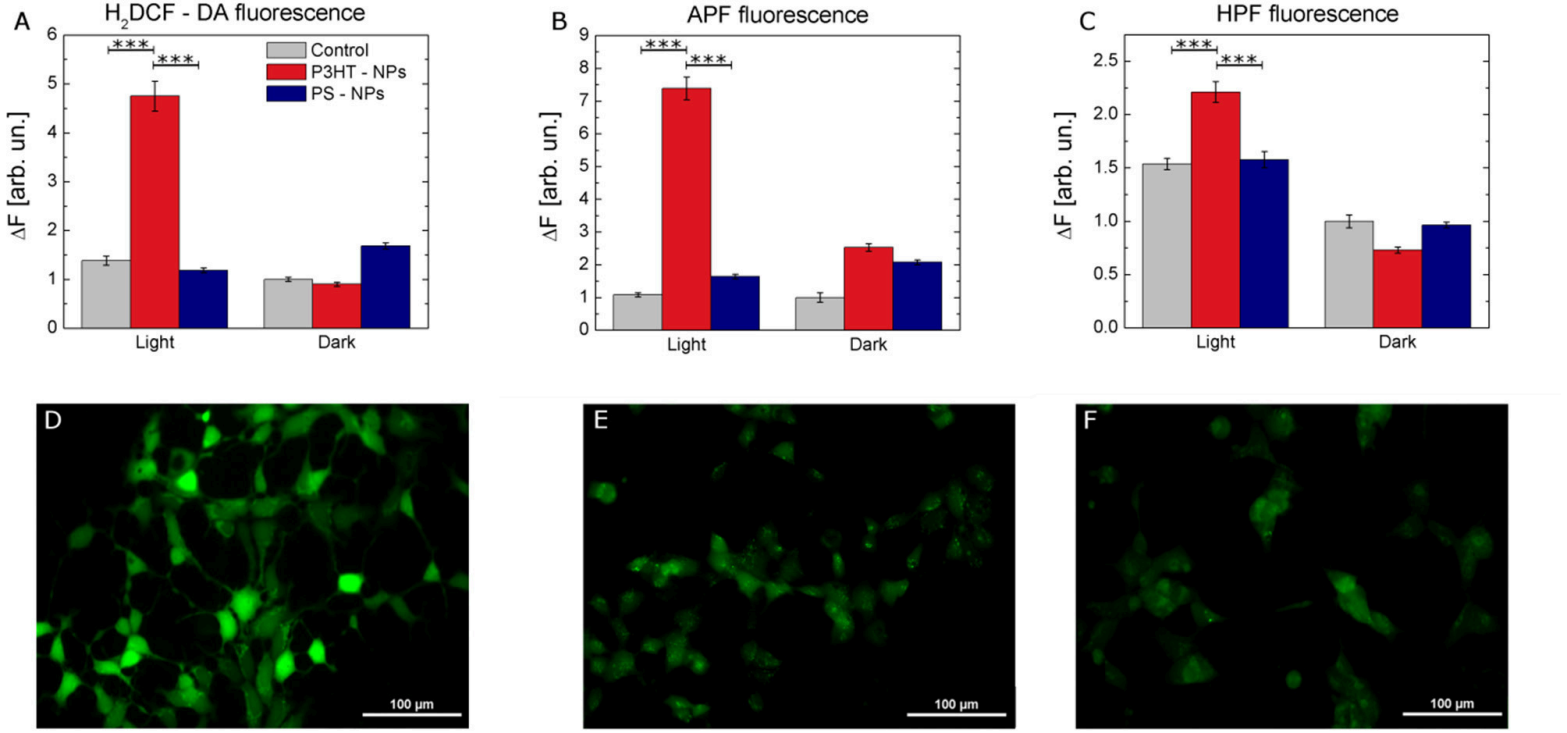
Intracellular ROS production by P3HT NPs optical excitation. **(A–C)** Fluorescence variation intensity measured in control (gray), P3HT NPs–treated (red), and PS NPs treated (blue) cells, due to ROS sensitive intracellular probes (H_2_DCF-DA, **A**, APF, **B**, and HPF, **C**). Data are represented as mean ± SE values, over statistical samples of *n* = 34 (control light), *n* = 81 (control dark), *n* = 49 (P3HT NPs light), *n* = 102 (P3HT NPs dark), *n* = 109 (PS NPs light), *n* = 105 (PS NPs dark) for H_2_DCF-DA; *n* = 57 (control light), *n* = 26 (control dark), *n* = 90 (P3HT NPs light), *n* = 63 (P3HT NPs dark), *n* = 70 (PS NPs light), *n* = 69 (PS NPs dark) for APF; *n* = 110 (control light), *n* = 118 (control dark), *n* = 137 (P3HT NPs light), *n* = 120 (P3HT NPs dark), *n* = 147 (PS NPs light), *n* = 120 (PS NPs dark) for HPF, where n represents the number of cells over three different experiments for each condition. Statistical significance has been evaluated by one-way ANOVA analysis followed by *post-hoc* Tukey test. *P*-values of the test are assigned as follows: ^***^ for *p* < 0.001. Statistically non-significant results are not marked in the figures. All details of statistical analysis are reported in the SI section. **(D-F)** Representative images of ROS-induced fluorescence response of H_2_DCF-DA **(D)**, APF **(E)**, HPF **(F)** in P3HT NPs treated cells. Fluorescence images have been acquired after carrying out the protocol for NPs excitation, as described in detail in the Methods section. Scale bar, 100 μm.

We experimentally compared the modulation of ROS production within the cell cytosol, in NPs treated and control samples, either in dark or subjected to photoexcitation. To this goal, we use intracellular ROS fluorescent probes, namely 2,7-dichlorodihydrofluorescein diacetate (H_2_DCF-DA), 2-[6-(4′-amino)-phenoxy-3H-xanthen-3-on-9-yl] benzoic acid (APF) and 2-[6-(4′-Hydroxy)-phenoxy-3H-xanthen-3-on-9-yl] benzoic acid (HPF). H_2_DCF-DA has been widely employed for the detection of ROS in several cell models, due to its capability to rapidly diffuse through the cellular membrane (Wang and Joseph, 1999). Cellular esterase first hydrolyzes the non-fluorescent H_2_DCF-DA to H_2_DCF, which is then oxidized by ROS and originates the fluorescent compound 2,7-dichlorofluorescein (DCF). Three different samples cohorts have been taken into account: non treated cells, cells treated with P3HT NPs, and cells treated with electrochemically inert, light-insensitive PS NPs. For each experimental group, two different conditions have been considered, cells subjected to illumination (2 min, 95 mW/mm^2^ photoexcitation density) prior to ROS detection analysis, and cells kept into dark conditions. We found that cells treated with NPs and subjected to illumination protocol show a statistically significant increase in ROS production, as compared to control untreated cells, and/or to treated cells in dark condition (Figure 3A). Since H_2_DCF-DA is sensitive to a large variety of different ROS, including H_2_O_2_, HO^.^, ROO^.^, we also used APF and HPF, which exhibit strong fluorescence enhancement upon O-dearylation, as induced by reactive species. APF and HPF provide complementary information with respect to H_2_DCF-DA because they are sensitive to HO^.^, ONOO^−^, and HOCl, but not to H_2_O_2_ (Gomes et al., 2005). In addition, they show much higher resistance to auto-photo-oxidation, being more suitable for the comparative evaluation of the effects of visible light illumination. APF and HPF data, shown in Figures 3B,C respectively, confirm that significant production of ROS occurs only in the case of cells treated with polymer NPs and previously subjected to the illumination protocol. Figures 3D–F show representative images of the fluorescence enhancement obtained in HEK-293 cells treated with P3HT NPs and illuminated for 2 min prior to ROS detection tests with H_2_DCF-DA (Figure 3D), APF (Figure 3E), and HPF (Figure 3F).

### Modulation of intracellular Ca^2+^ dynamics

Recent literature highlighted the mutual crosstalk between ROS and Ca^2+^ dynamics. It has been reported that the cell redox state can efficiently modulate the activity of a variety of Ca^2+^ channels, and, on the other hand, Ca^2+^ signaling actively contributes to control the production of ROS^47^.

To determine if the observed ROS enhancement leads to specific modulation of calcium dynamics, we monitored the intracellular Ca^2+^ ion concentration by using Fluo-4 as a calcium indicator. We employed two different light photoexcitation densities, namely 2.1 and 14.6 mW/mm^2^.

Taking advantage of the partial spectral overlap between the light absorption of the polymer NPs and the excitation spectrum of the Fluo-4 calcium indicator, we used the same illumination protocol both to optically excite the P3HT NPs and to monitor Ca^2+^ dynamics at the same time. In all considered cases continuous illumination for 3 min has been employed, coherently with protocols used in photoelectrochemical measurements and ROS detection analysis.

**Figure 4:**
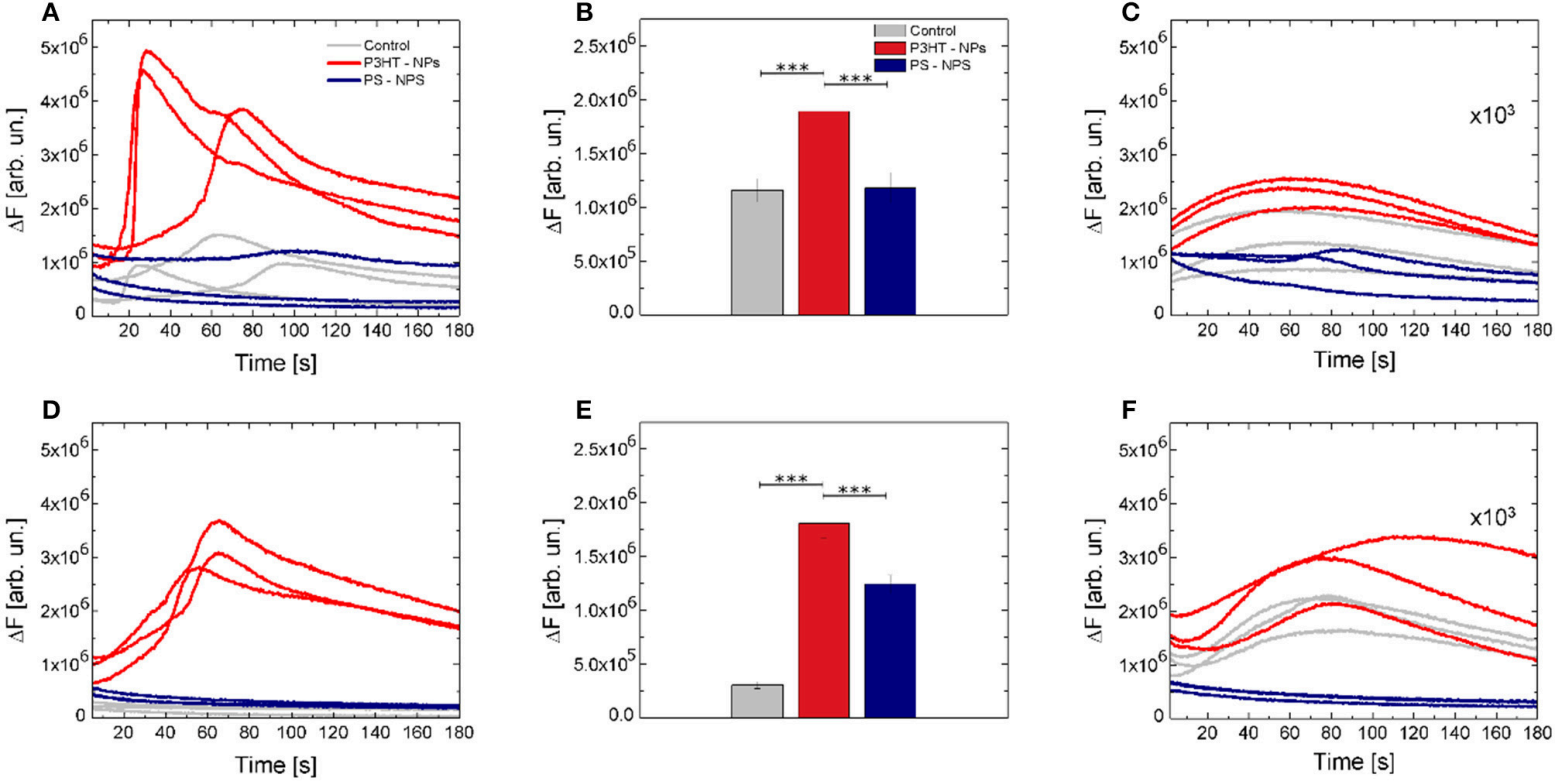
Optical modulation of intracellular Ca^2+^ dynamics. **(A)** Representative Ca^2+^ dynamics recorded in control (gray solid lines), P3HT NPs treated cells (red solid lines) and PS NPs treated cells (blue solid lines). **(B)** Fluorescence relative increase of the Fluo-4 calcium indicator. Data are shown as mean ± SE, over statistical sample sets of *n* = 71, *n* = 68, *n* = 55 cells (for control, P3HT NPs and PS-NPs samples, three experiments for each condition, respectively). Statistical analysis has been carried out by one-way ANOVA, followed by *post-hoc* Tukey test. *P*-values of the test are assigned as follows: ^***^ for *p* < 0.001. Statistically non-significant results are not marked in the figure. See SI section for all details of statistical analysis. **(C)** Effect of the specific ROS inhibitor (NAC) on Ca^2+^ dynamics. Data have been multiplied by a 10^3^ factor, to make them visible over the same y-axis scale used in **(A)**. **(D–F)** Representative Ca^2+^ dynamics, Fluo-4 fluorescence relative increase and effect of NAC ROS inhibitor, in absence of extracellular Ca^2+^ ions. In panel **E**, data are shown as mean ± SE, over statistical sample sets of *n* = 29, *n* = 60, *n* = 37 cells (for control, P3HT NPs and PS-NPs samples, respectively).

While optical stimulation at low excitation density (2.1 mW/mm^2^) did not lead to significant differences among considered experimental conditions (Figures S7A,D,G), higher intensity (14.6 mW/mm^2^) optical excitation of P3HT NPs -treated cells led to significant activation of Ca^2+^ dynamics (Figure 4). Figure 4A shows 3 representative Ca^2+^ transient dynamics for each considered case; the whole ensemble of registered curves (*n* ≥ 100) is reported in Figures S7B,E,H. Ca^2+^ peak amplitude shows a relative increase of about 60% both with respect to untreated cells and to cells treated with PS NPs. No significant changes in Ca^2+^ dynamics were detected in positive and negative control experiments, i.e., in illuminated, untreated cells and illuminated cells treated with inert, light-insensitive PS NPs (Figure 4B). Representative fluorescence images at different time points are reported in Figures S12A–H, to give a direct reply of the reported curves.

The Ca^2+^ sources involved in the stimulation, e.g., intracellular stores and/or extracellular environment, were investigated in Ca^2+^-free conditions, and in the presence of 5 mM of the Ca^2+^ chelator ethylene glycol tetraacetic acid, EGTA. Figure 4D shows 3 representative curves for each case. The whole data set is reported in Figures S8A,D,G at lower intensity photoexcitation and Figures S8B,E,H at a higher intensity photoexcitation. Representative fluorescence images at different time points are reported in Figures S12I–R, to give a direct reply of the reported curves. Figure 4E shows that the relative increase of fluorescence intensity exhibited by P3HT NPs-treated cells in absence of extracellular Ca^2+^ is not significantly different from the one obtained in standard conditions (ΔF = 1.92 and ΔF = 1.85, respectively). This indicates that Ca^2+^ influx through the plasma membrane does not play a significant role and that the main origin of activation is in intracellular Ca^2+^ stores.

Based on results reported so far, we conjecture that light-evoked production of ROS in the cell cytosol acts as the main source of the observed intracellular Ca^2+^ increase. In order to verify this hypothesis, after cell cultures illumination, we supplemented cell cultures with a well-known ROS inhibitor, N-Acetyl-L-cysteine (NAC), and thereafter we carried out Ca^2+^ fluorescence imaging experiments, reported in Figures 4C,F (complete data sets are shown in panels **C,F,I** of Figures S7, S8). Interestingly, Ca^2+^ dynamics were significantly silenced, in the presence of extracellular Ca^2+^ as well as in Ca^2+^ free extracellular solution.

### Dependence of ROS production and Ca^2+^ modulation on P3HT NPs concentration

**Figure 5:**
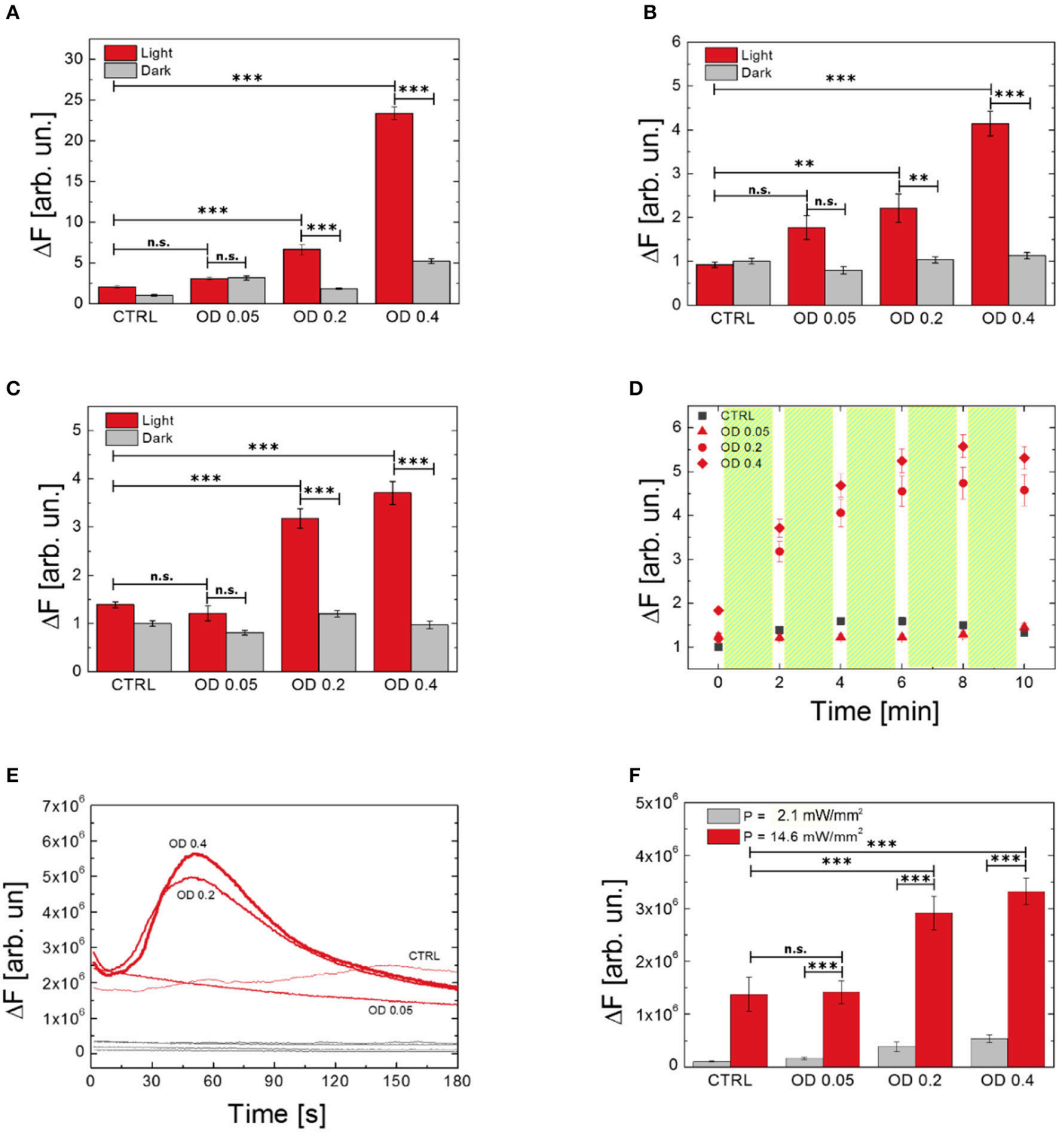
Effect of P3HT NPs dispersion concentration on ROS generation by using different probes (**A**, H_2_DCF-DA; **B**, HPF; **C,D**, APF) and Ca^2+^ modulation **(E,F)**. Data report the mean values over populations, three experiments for each condition, of N_H2DCF−DA_Light_OD0.4_ = 66, N_H2DCF−DA_Dark_OD0.4_ = 40, N_H2DCF−DA_Light_OD0.2_ = 46, N_H2DCF−DA_Dark_OD0.2_ = 40, N_H2DCF−DA_Light_OD0.05_ = 48, N_H2DCF−DA_Dark_OD0.05_ = 30, N_H2DCF−DA_Light_untreated_ = 42, N_H2DCF−DA_Dark_untreated_ = 30, N_HPF_Light_OD0.4_ = 48, N_HPF_Dark_OD0.4_ = 46, N_HPF_Light_OD0.2_ = 48, N_HPF_Dark_OD0.2_ = 43, N_HPF_Light_OD0.05_ = 54, N_HPF_Dark_OD0.05_ = 52, N_HPF_Light_untreated_ = 47, N_HPF_Dark_untreated_ = 39, N_APF_Light_OD0.4_ = 56, N_APF_Dark_OD0.4_ = 56, N_APF_Light_OD0.2_ = 69, N_APF_Dark_OD0.2_ = 69, N_APF_Light_OD0.05_ = 48, N_APF_Dark_OD0.05_ = 48, N_APF_Light_untreated_ = 42, N_APF_Dark_untreated_ = 42, N_FLUO−4_OD0.4_ = 43, N_FLUO−4_OD0.2_ = 51, N_FLUO−4_OD0.05_ = 60, N_FLUO−4_untreated_ = 56. **(D)** Shows the effect on ROS production of repeated light stimulation events, each one of 2 min overall duration, as evidenced by using the APF probe. Statistical analysis has been carried out by one-way ANOVA, followed by *post-hoc* Tukey test. *P*-values of the test are assigned as follows: ^***^for *p* < 0.001, ^**^for *p* < 0.005. Statistically non-significant results are marked as n.s. in the figures. See SI section for all details of statistical analysis.

We investigated the effect of different polymer NPs dispersion concentrations on ROS production and modulation of Ca^2+^ dynamics (Figure 5). We compared three concentrations of NPs, corresponding to optical densities of 0.05, 0.2, and 0.4. Figures 5A–C show the effect of different NPs doses on ROS production, as evaluated by using H_2_DCF-DA, HPF, and APF probes, respectively. In all the considered cases, the increased number of internalized P3HT NPs does not influence, *per se*, the production of ROS, which is not statistically different from the values recorded in untreated, control samples (dark condition, gray histograms). Conversely, upon visible light excitation, NPs concentration strongly affects ROS evolution (light condition, red histograms). We notice that the lowest NP concentration, corresponding to an initial optical density of the dispersion of 0.05, does not lead to a substantial increase of ROS production, thus representing a lower limit for obtaining sizable physiological effects. Conversely, as expected, an increasingly higher number of NPs leads to enhanced photogeneration of charges, thus determining an overall, sizable increase of ROS production. It is worth noting how the enhancement of ROS in dependence on NPs concentration is more pronounced in the case of the H_2_DCF-DA probe, being the latter sensitive to several species. Conversely, the response of APF and HPF probes presents a smoother dependence on NPs concentration with respect to H_2_DCF-DA, probably due to their higher selectivity. Interestingly, Figure 5D shows the temporal dynamics of ROS evolution upon a photostimulation pattern (2 min photoexcitation followed by 10 s in dark, repeated five times), as detected by the APF probe. After three photoexcitation events, the ROS production reaches a saturation concentration. We conclude that the main effect is achieved within the first minutes of photostimulation. We carried out the same experiment using H_2_DCF-DA and HPF probes, but unfortunately, these probes undergo photo-oxidation and bleaching phenomena upon prolonged illumination, which makes them unreliable for this kind of study.

Ca^2+^ dynamics as well are heavily affected, upon visible light excitation, by the concentration of P3HT NPs. Intracellular Ca^2+^ dynamics and averaged maximum fluorescence variation at different NPs concentrations and two photoexcitation densities (2.1 and 14.6 mW/mm^2^) are shown in Figures 5E,F, respectively. In line with results obtained for ROS production, the lowest employed NPs concentration, corresponding to an initial optical density of the dispersion of 0.05, does not lead to sizable effects. The Ca^2+^ response upon photostimulation density of 2.1 mW/mm^2^ is fully comparable to the physiological Ca^2+^ concentration, as obtained in control samples. Conversely, effective NPs excitation, with a photoexcitation density of 14.6 mW/mm^2^, leads to a pronounced modulation of Ca^2+^ dynamics. Overall, these data show that both ROS and Ca^2+^ increase strongly depends on the concentration of P3HT NPs, and further demonstrate the key-role of polymer charge generation upon visible light.

### Photo-toxicity assays

**Figure 6:**
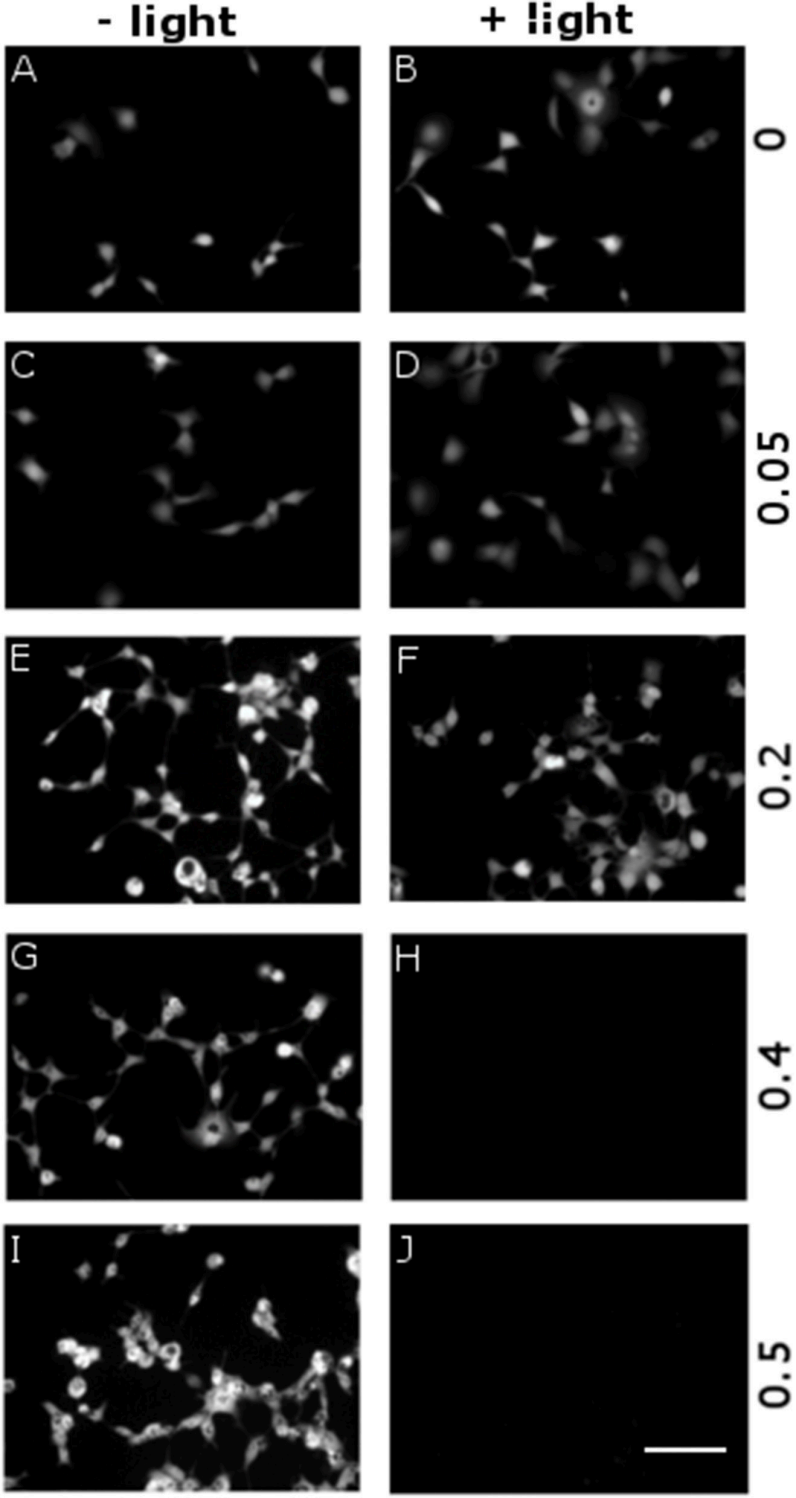
Dependence of cytotoxicity on P3HT NPs dose and optical excitation. P3HT NPs at different concentrations, corresponding to OD in the range 0–0.5 (from top to bottom panels), have been administered to HEK-293 cells at the plating step. A sub-set of samples has been treated with the same illumination protocol used for ROS production (CW LED light illumination, peak emission wavelength λ = 540 nm, closely matching the polymer absorption spectrum; photoexcitation density P = 95 mW/mm^2^; overall duration of the photoexcitation protocol, 2 min), 24 h after NPs administration and upon careful rinsing of the cell growth medium **(B, D, F, H, J)**. All other samples have been subjected exactly to the same preparation protocol, but they did not undergo photoexcitation **(A, C, E, G, I)**. Viable cells are finally detected by FDA staining. Representative images have been acquired at 2 DIV. Scale bar, 100 μm.

We evaluated the cytophototoxicity effects eventually determined by optical excitation of polymer NPs, as compared with samples treated with NPs but not subjected to the illumination protocol, i.e., in which no ROS generation was optically activated. We considered five NPs doses, corresponding to OD in the range 0–0.5 (0, 0.05, 0.2, 0.4, and 0.5 OD values). Photoexcitation was carried out 24 h after cell plating/NPs administration, by using the same protocol employed for inducing ROS generation (see Figure 5 and related methods). In all cases (i.e., for both illuminated and non-illuminated samples) cell viability was evaluated 48 h after plating and NPs administration. Figure 6 displays representative images obtained by fluorescein diacetate (FDA) assay. FDA is a non-fluorescent molecule, which is hydrolyzed to fluorescent fluorescein in live cells only. We notice that cells not subjected to the illumination protocol show good viability at all considered NPs doses (Figures 6A,C,E,G,I). In the case of illuminated samples (Figures 6B,D,F,H,J), photoexcitation of P3HT NPs leads to substantial toxicity effects at the two highest NPs concentrations considered here (Figures 5H,J). Conversely, at P3HT NPs doses corresponding to 0–0.2 OD values no remarkable toxicity effect is detected. By combining these results with data reported in Figure 5, we identify the intermediate NPs concentration, corresponding to OD 0.2, as the most suitable one, leading to effective ROS production and reliable Ca^2+^ ions modulation, but with no detrimental effects on cell viability, as evaluated 24 h after photoexcitation.

**Figure 7:**
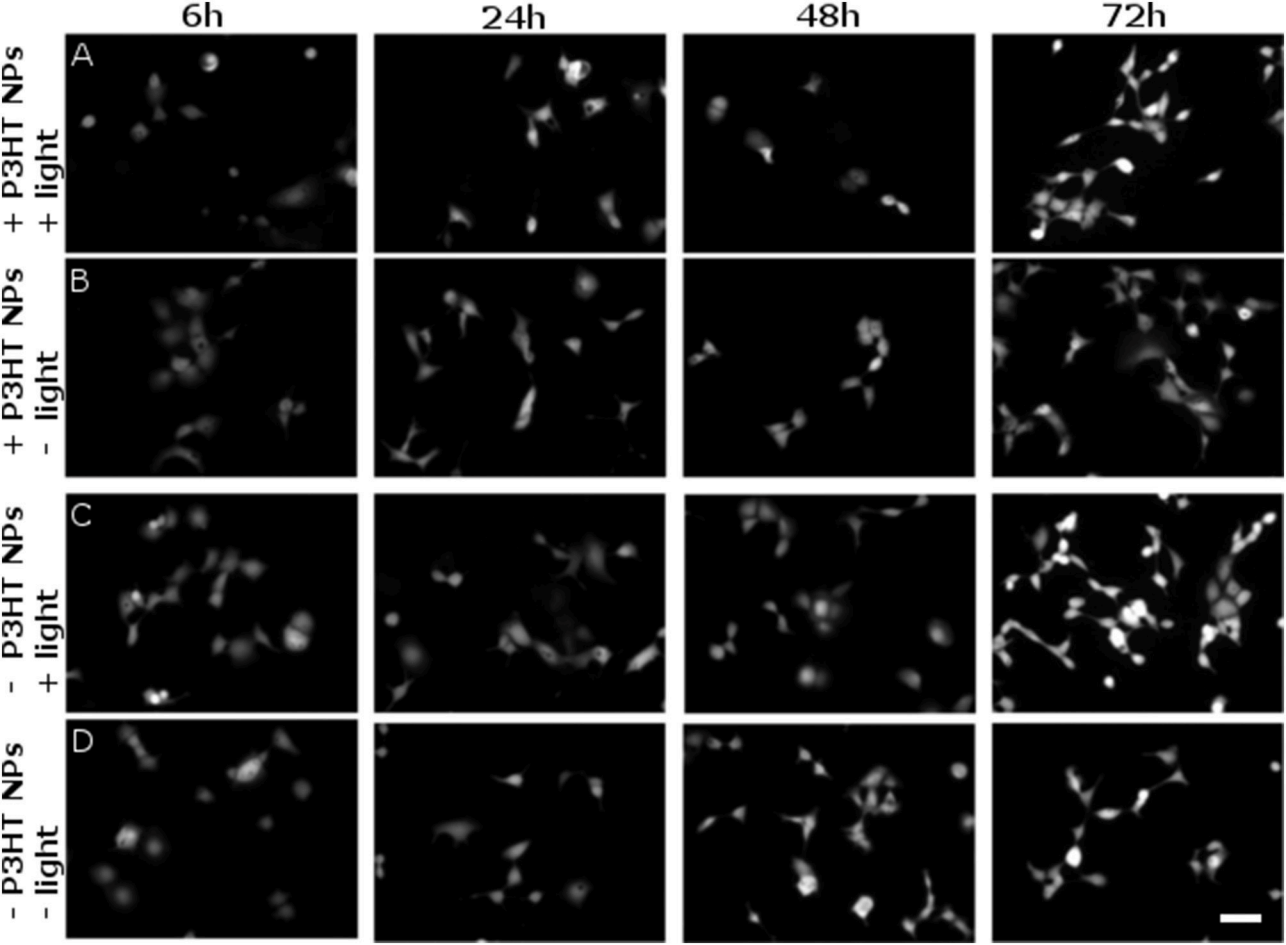
Evaluation of delayed cytotoxicity effects. Cell viability has been tested by FDA staining assay, in presence and absence of P3HT NPs (± P3HT NPs, 0.2 OD, panel lines **A–D** respectively), and in presence or absence of polymer photoexcitation (± light, panel lines **A–D**, respectively), at fixed time points after plating and NPs administration. At 1 DIV, the same illumination protocol used for ROS generation was applied to samples treated by light (CW LED light illumination, peak emission wavelength λ = 540 nm, closely matching the polymer absorption spectrum; photoexcitation density P = 95 mW/mm^2^; overall duration of the photoexcitation protocol, 2 min). Viable cells were then stained at fixed time points, namely 6, 24, 48, 72 h after the illumination protocol, corresponding to a maximum of 4 DIV (from left to right panels, respectively). Scale bar, 100 μm.

However, it is known that ROS can also lead to delayed toxicity effects, visible only after few days after production. Thus, we carried out viability tests at different time points, up to 72 h after NPs photoexcitation (at 4 DIV) and related ROS generation. Figure 7 shows representative images of viable cells stained by FDA. The comparison among 4 different sample cohorts (NPs treated/untreated and illuminated/non-illuminated) does not show significant differences in the staining of viable cells. Most importantly, no remarkable signs of delayed photocytotoxicity effects are evidenced.

## Discussion

In this work, we study the coupling mechanism at the abiotic/biotic interface between polymer NPs and living cells upon photoexcitation with visible light. Our NPs are good candidates for light-driven cell actuation. Indeed, functional effects on HEK-293 cells electrophysiology and in *Hydra Vulgaris* have been recently demonstrated (Tortiglione et al., 2017; Zangoli et al., 2017). Here, experimental data demonstrate that optical excitation of polymer NPs enhances the physiological ROS production and elicits intracellular Ca^2+^ dynamics. We propose that this is occurring to a suitable extent to cause physiological effects and yet it is below the phototoxic regime.

First, we observe that upon photoexcitation with visible light P3HT NPs suspended into an aqueous electrolyte generate a photocurrent signal. We assign this to redox processes, accordingly to what occurs in P3HT films when used as the light-sensitive working electrode in photo-electrochemical cells. The same process is invoked to explain the observed generation of ROS in the cytosol compartment within living cells.

Several inorganic NPs, including metallic NPs (Wang et al., 2017), inorganic nanocrystals (Ipe et al., 2005; Wang et al., 2015) and magnetic NPs, have been reported to have sizable effects on the ROS population, thus representing an interesting strategy for photothermal therapies (Ye and Chen, 2016; Dayem et al., 2017). Among carbon-based materials, carbon nanotubes, graphene and polymer nano-bioconjugates can also sustain photo-thermal stimulation (Miyako et al., 2014; Chechetka et al., 2016; Lyu et al., 2016; Li et al., 2017). Yet, there is a fundamental difference to be taken into account when comparing those systems with our P3HT NPs: for both inorganic- and organic-based systems reported so far, NIR-mediated photo-thermal heating is the main phenomenon, *indirectly* leading to ROS enhancement. Conversely, in our case, significant thermal effects can be ruled out. In fact, for a single NP upon light excitation density of I_0_ = 100 mW/mm^2^ the expected temperature increase is ΔT = I _0_ π ${\text{R}}_{\text{NP}}^{2}$ / (4 π K r), where R_NP_ = 240 nm is the average NP radius, K = 0.6 W/ mK is the thermal conductivity of water and r is the distance from the NP center. At the particle surface (r = R_NP_), the expected ΔT is in the order of 10 mK, a value considered too low for provoking sizable effects. In order to directly test the effect of NPs-mediated thermal increase also on an experimental ground, we carried out electrophysiology experiments (whole cell patch clamp configuration) in HEK-293 cells stably transfected with temperature-sensitive channels (TRPV1), a system which intrinsically bears enhanced sensibility to temperature variations. Upon photoexcitation of P3HT NPs internalized within the cell cytosol we could not observe any significant effect (Figure S9) mediated by TRPV1 channel response. This confirms the numerical evaluation and rules out the thermal effect as a primary result of photoexcitation.

We conclude that the observed changes in ROS concentration and Ca^2+^ dynamics are most plausibly caused by a direct photocatalytic effect. The dependence of the cell response on internalized NPs concentration and light excitation protocol further supports this hypothesis (Figure 5).

It is important to notice again that as soon as the delicate balance of ROS production is altered, or the intracellular levels of antioxidants is lowered, ROS can become highly harmful, causing oxidative stress which, in some cases, leads to irreversible cell damage (Martindale and Holbrook, 2002). Based on a detailed dose-response analysis and cell viability tests, we identify a suitable NPs concentration, corresponding to 0.2 OD value of the colloidal dispersion as an optimal compromise between reliable functional photomodulation of ROS generation and intracellular Ca^2+^ ions increase, on one side, and occurrence of cytophototoxicity effects on the other side (Figure 6). In these conditions, and within the temporal window considered in the present work, up to 4 DIV (compatibly with the use of *in vitro* HEK-293 cell model), we do not observe sizable toxicity effects within treated cells, as documented by fluorescence imaging before and immediately after the photoexcitation protocol (Figure S10), as well as at different time points after photoexcitation, up to 72 h (Figure 7).

**Figure 8:**
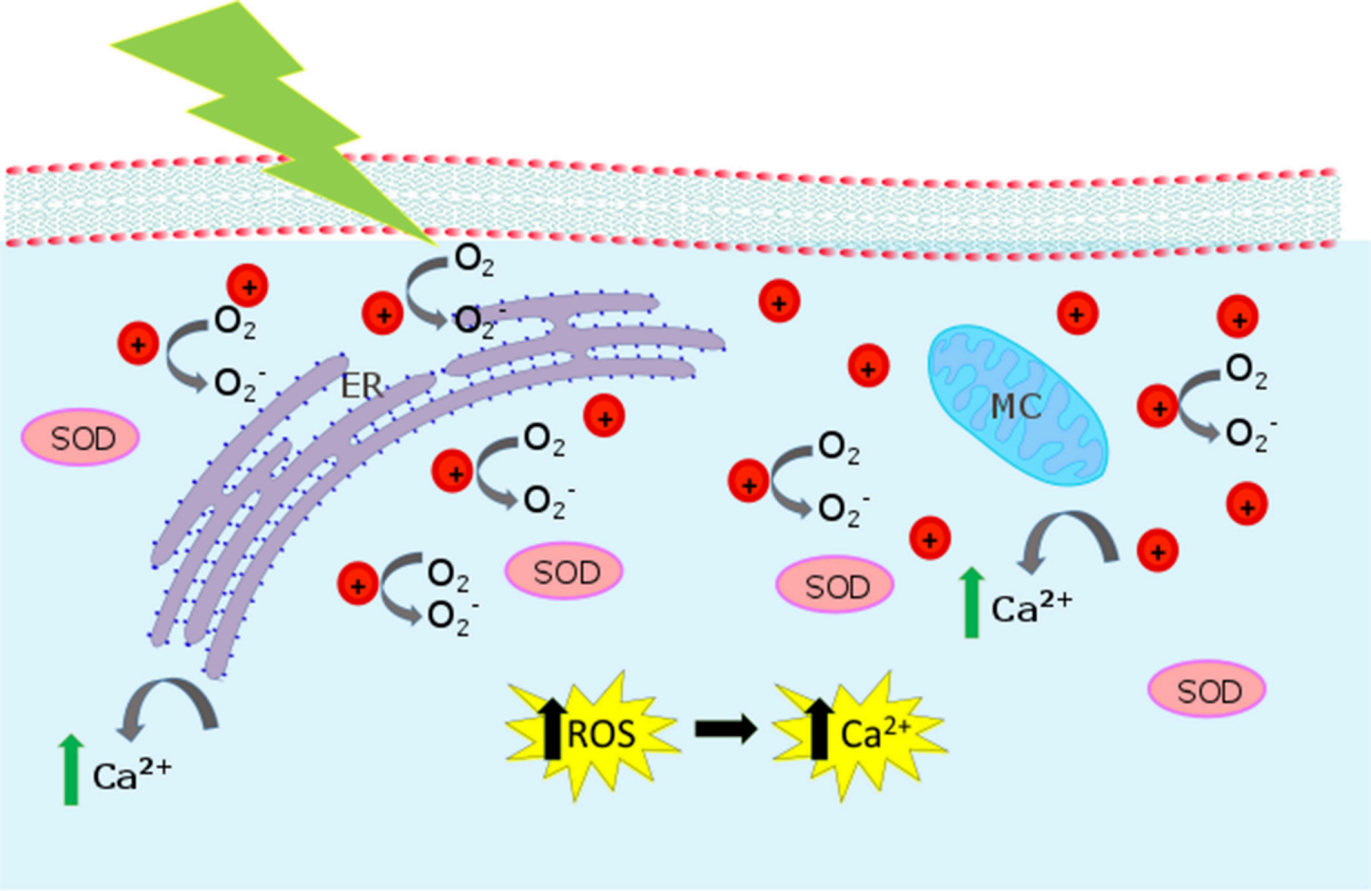
Biological pathways activated by P3HT-NP photostimulation. The cartoon represents the intracellular compartment, showing mitochondria (MC) and endoplasmic reticulum (ER). When P3HT NPs are irradiated, ${\text{O}}_{2}^{-}$ is produced. ${\text{O}}_{2}^{-}$ is expected to act as an intermediate toward the formation of other ROS and to increase the SOD (superoxide dismutase) concentration. Increased Ca^2+^ release from intracellular sources is also observed.

The proposed interaction mechanisms leading to ROS enhancement are depicted in Figure 8, based on the well-known P3HT photo-physical scenario. P3HT belongs to the class of polymeric organic semiconductors. It has a carbon conjugated backbone that supports π-electron delocalization. In solution, as isolated chains, such polymers behave like large organic molecules. The main photo-excited species are singlet and triplet states, the latter mostly produced by intersystem crossing from the initial singlet. Due to the extended conjugation, a fraction of the initial singlet states may dissociate into charge pairs. Those are very short-lived (< 1 ps) in isolated chains, but in a solid, where inter-chain coupling may be non-negligible, they can separate on different chains and survive up to the milliseconds' timescale. In presence of oxygen (or any other dopants) this scenario dramatically changes. The singlet state quenches at the dopant, by energy or charge transfer. In the latter case, this generates fairly mobile holes and trapped electrons, a behavior often referred to as p-type. P3HT has a remarkably small triplet photogeneration yield (below 0.1%) (Jiang et al., 2002). This has important implications for biophotonics, since it substantially reduces the photo-toxicity of the material, cutting down singlet oxygen generation, the major killing path in photodynamic therapy. Accordingly, we think that optical excitation of the polymer NPs leads to singlet and polaron (charged) states. Singlets or negative polarons react with the oxygen dissolved in the aqueous dispersion (Figure 2) or in the cytosol environment, first reducing oxygen. The superoxide further evolves leading to a generation of different ROS (Figure 3). Because we use CW light, the steady state population of the singlet state is small (the lifetime is in the order of 1 ns). For this reason, the polaron-mediated path seems the most plausible one.

Polymer-mediated production of ROS deterministically triggers an increase in the intracellular Ca^2+^ concentration, as demonstrated by the fact that upon ROS inhibition by a selective pharmacological agent (NAC), variations in Ca^2+^ dynamics are completely suppressed (Figure 4). Moreover, we evidence that the main source of intracellular Ca^2+^ increase resides within the cytosol, thus indicating a crucial role in the Ca^2+^ release by mitochondria and/or the endoplasmic reticulum, being these organelles mostly involved in Ca^2+^ dynamics. Further studies would be needed, however, to exactly identify the organelle involved in the photo-transduction effect triggered by illuminated P3HT NPs, and also to assess if the Ca^2+^ pathway on the cell membrane is eventually changed.

Both Ca^2+^ ions and ROS represent prominent signaling pathways able to finely tune several cell functions (Chakraborti et al., 1999). Importantly, dysfunctions of the delicate equilibrium in Ca^2+^/ROS balance have been reported to have serious implications in various disorders, including, among others, neurodegenerative diseases such as Parkinson's and Alzheimer's disease, inflammatory diseases, metabolic diseases, and ischemic events (Chinopoulos and Adam-Vizi, 2006; Chaudhari et al., 2014; Sharma and Nehru, 2015; Zucchetti et al., 2016). In perspective, our results represent a novel, minimally invasive and locally confined tool for efficient modulation of ROS and intracellular Ca^2+^ concentration.

## Author contributions

CB carried out all the experimental measurements, with help from IA (electrochemical characterization, ROS measurements, Ca^2+^ Dynamics), GT (electrochemical characterization). EZ fabricated P3HT NPs, DD carried out TEM experiments. MZ and FD synthesized P3HT for the NPs. MA and GL planned and supervised the research. All authors contributed to manuscript drafting and approved the final version of the manuscript.

### Conflict of interest statement

The authors declare that the research was conducted in the absence of any commercial or financial relationships that could be construed as a potential conflict of interest.
