# Supplementary material for: Photocatalytic Activity of Polymer Nanoparticles Modulates Intracellular Calcium Dynamics and Reactive Oxygen Species in HEK-293 Cells
Source: Front Bioeng Biotechnol. 2018 Aug 23;6:114. doi: 10.3389/fbioe.2018.00114 (PMC6119808; doi:10.3389/fbioe.2018.00114)
Supplement: Supplementary file 1 [file Data_Sheet_1.pdf]

Supplementary Material

Photocatalytic activity of polymer nanoparticles modulates intracellular calcium dynamics and reactive oxygen species in HEK-293 cells

Caterina Bossio [1], Ilaria Abdel Aziz [1, 2], Gabriele Tullii [1, 2], Elena Zucchetti [1, 2], Doriana Debellis [3], Mattia Zangoli [4], Francesca Di Maria [4], Guglielmo Lanzani [1, 2], Maria Rosa Antognazza [1, *]

[1] Center for Nano Science and Technology@PoliMi, Istituto Italiano di Tecnologia, via Giovanni Pascoli 70/3, 20133 Milano, Italy
[2] Dip.to di Fisica, Politecnico di Milano, P.zza L. da Vinci 32, 20133 Milano, Italy
[3] Electron Microscopy Facility, Istituto Italiano di Tecnologia, Via Morego 30, 16163 Genova, Italy
[4] Institute for Organic Synthesis and Photoreactivity, CNR-ISOF, via P. Gobetti 101, 40129 Bologna, Italy

[*] Corresponding author:
Maria Rosa Antognazza
Via Pascoli 70/3, 20133 Milano, Italy
Tel.: +39 02 2399 9881
e-Mail: Mariarosa.antognazza@iit.it

P3HT NPs Characterization

Morphological, optical and stability properties

Figure S1A shows Dynamic Light Scattering (DLS) measurements of P3HT NPs dispersion, in aqueous solution. We obtain an average hydrodynamic diameter (HD) of 237 ± 82 nm, and a polydispersity index of 0.12. The high negative Z-potential value (-35 ± 8 mV) observed in aqueous environment indicates the presence of a negative charge on mobile solvent surface, leading to intense electrostatic repulsion between NPs, which indicates good colloidal stability and prevents NPs massive flocculation and precipitation. NPs colloidal stability over time, up to 48 hours, has been evaluated by carrying out both DLS measurements, by assessing modifications of the hydrodynamic diameter (HD) values, and UV-Vis spectroscopy, by evaluating changes in the optical absorbance and photoluminescence spectra. Importantly, since it is well-known that counter-ions can screen the NP charge and cause overall instability of colloidal properties (Hühn et al., 2013; Puertas et al., 1999), NPs were dispersed within the cell culturing medium (D-MEM, without phenol red). HD values, normalized to the initially recorded value (at time 0), are shown in panel B. Measurements have been performed by employing different initial optical densities of the NPs dispersion, in the range 0.2 – 0.5, in line with NPs dispersion concentration administered to living cells cultures. Only slight variation (<10%) with respect to the initial HD value is observed in the considered, operating conditions. Normalized optical absorbance spectra are shown in panel C, at considered time points. One should notice that the spectral features of the optical absorption, peculiar of P3HT NPs, do not show significant changes over time, thus indicating good stability of P3HT NPs within the cell culturing medium and negligible aggregation effects. Importantly, no sizable red-shift of the absorption peak is detected. The good stability of the dispersion is also confirmed by photoluminescence spectra (panel D). As expected for P3HT NPs in aqueous solution, the photoluminescence efficiency is progressively quenched, but no changes in the spectrum profile are evidenced. Overall, reported data show very good colloidal stability of the NP suspension in the cell culturing medium, up to 48 hours.

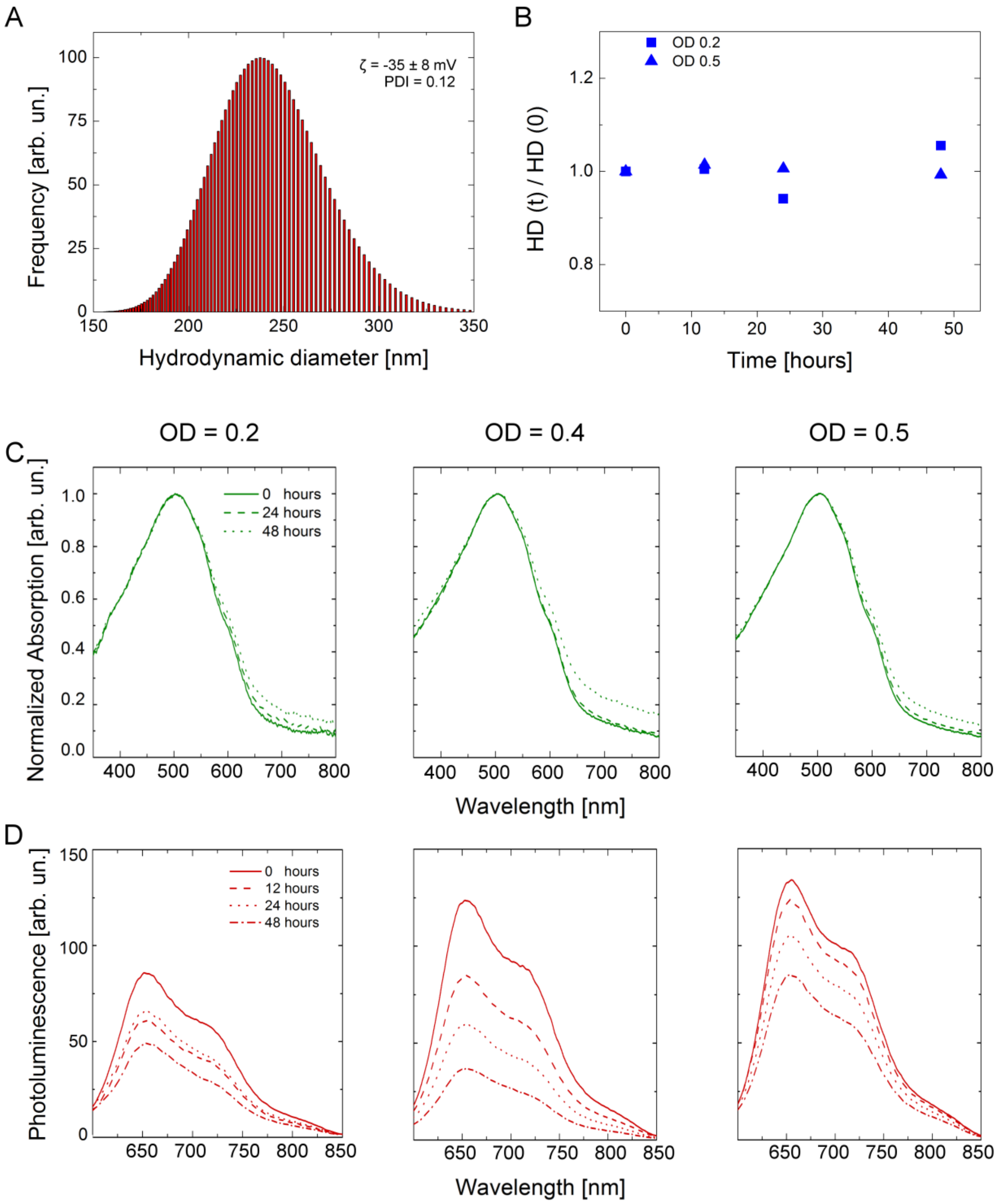

Fig. S1. (A) Dynamic Light Scattering measurements of P3HT NPs water dispersion. The average hydrodynamic diameter (HD) is 237 ± 82 nm, the polydispersity index (PDI) is 0.12. Standard deviation (SD) of the HD values has been calculated according to the relationship $PDI = (SD/HD)^2$. (B) Colloidal stability tests in complete cell culturing medium (D-MEM +10% FBS). Relative variation of the HD, normalized to the initial value at time 0, is shown. Two NPs concentrations have been considered, corresponding to initial optical densities 0.2 and 0.5 (square and triangles symbols, respectively). (C), (D) Temporal evolution of optical absorption (C) and photoluminescence spectra (D) of NPs dispersed within the cell culturing medium, at fixed initial optical densities (0.2, 0.4 and 0.5 shown in left, middle and right panels, respectively).

Cytotoxicity test

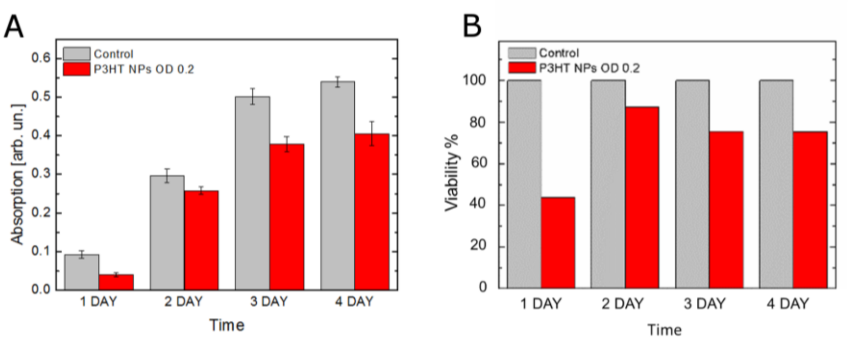

Fig. S2. (A) MTT viability assay on P3HT NPs –treated HEK-293 cells and on control, untreated samples. P3HT NPs dispersion (OD 0.2) was administered alongside with cells plating. Data are reported as average over N = 5 samples ± standard error of the mean. (B) Viability percentage variation shows that cell toxicity effects induced by P3HT NPs are progressively quenched. Starting from DIV 2, treated cells display reduced viability of about 20% as compared to control, untreated samples.

Evaluation of P3HT NPs upload within the cell cytosol

The uptake of P3HT NPs within the cell cytosol has been evaluated by using two different methods, the first one based on fluorescence imaging (Fig. S3) and the second one on the variation of the optical absorption of the dispersion before and after administration to the cell culture for 24 hours (Fig. S4). Three initial concentrations of the dispersion have been considered, corresponding to optical densities of 0.05, 0.2 and 0.4. Fluorescence images show that NPs are both internalized by the cells and docked at the glass substrate. The evaluation has been carried out on regions of interest only comprising areas covered by cells. In this way, the final computation takes into account only the nanoparticles internalized within the cells. The whole field of view contained both cells and uncovered glass: region of interest of the exact shape of cells were chosen, in order to carry out the calculation only over the cell volume. The analysis of these regions was carried out with a dedicated macro running on ImageJ software, able to measure particles' size and number for each region of interest. Figure S3 reports the calculated number and radii distribution of NPs (Panels A-C) for the three different employed optical densities, the number being related to the whole population in the regions of interest. Data show that 80% of the counted nanoparticles have radii around 300 nm. Upon summing over all counted NPs, we estimate that, in the case of initial OD 0.2 and 0.4, a few hundreds of NPs/cell have been internalized within the cytosol. In the case of lower initial dispersion concentration (OD 0.05), a considerably lower fraction of NPs undergoes efficient internalization (Panel D).

In order to confirm this rough estimation, optical absorption measurements, at time 0 (corresponding to NPs administration, at the working concentration) and after 24 hours, have been carried out (Fig. S4). As described in the Methods section, the NPs are administered to the cells by diluting them in the extracellular medium alongside with the cell plating step. Some of them dock on the glass substrate and some of them are internalized, therefore it is possible to record an absorbance variation with respect to the initial concentration of NPs. Figure S4 reports the absorption spectrum of the initial dispersion (black solid line) and the one after 24 hours of incubation (red solid line). The absorbance variation has been evaluated in the spectral region represented in Figure S4 as a blue rectangle, centered at $\lambda = 506 \pm 3$ nm. We notice that the variation obtained in the case of initial middle (OD 0.2) and higher (OD 0.4) concentration is comparable, about 1%, and definitely higher than the one recorded for the lowest initial concentration (about 0.05%, for OD 0.05). Numerical results are reported below. The 1% variation corresponds, by taking into account the confluence level of cultured cells, to an average number of internalized NPs of about 500/cell, which, in turn, is possibly overestimated, due to the fact that in this case NPs docked to the Petri culturing dish cannot be taken into account.

Overall, these two methods provide a rough estimation of the effective NP cell loading. These results are in line with measurements of ROS and intracellular Ca^{2+} dynamics at different NPs dispersion concentration (see main text, Figure 5).

	**OD 0.05**	**OD 0.2**	**OD 0.4**
Absorbance variation	5.1×10^{-4}	1.1×10^{-2}	0.8×10^{-2}

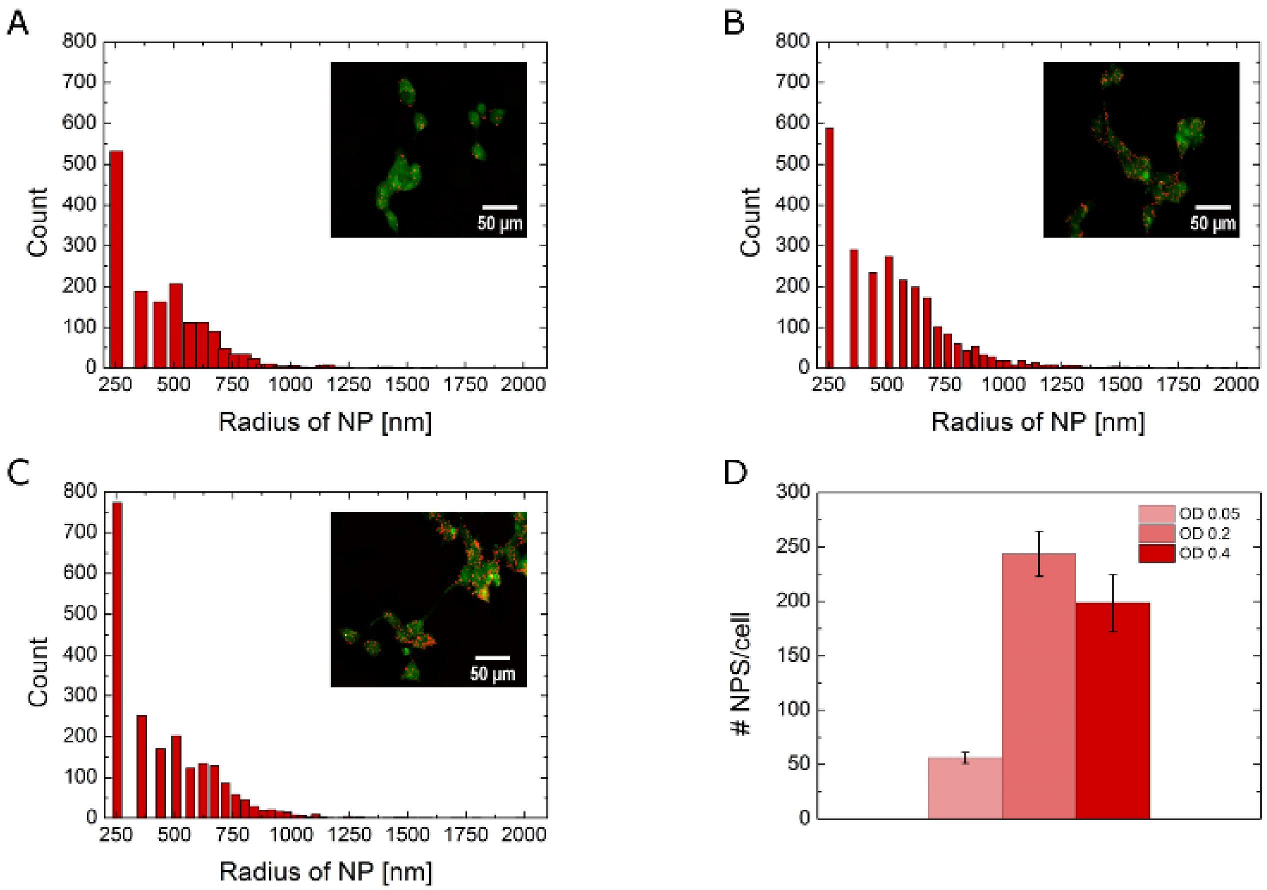

Fig. S3. Average distribution of P3HT NPs radii, internalized within HEK-293 cells, as obtained by fluorescence imaging analysis. Cell cytosol is stained by phalloidin-FITC (green), P3HT NPs intrinsic photoluminescence is visible in the red channel. (A-C) Three different initial concentrations, corresponding to optical densities of 0.05 (panel A), 0.2 (panel B) and 0.4 (panel C), are considered. Insets show representative fields of view. (D) Estimated average number of NPs within a single cell.

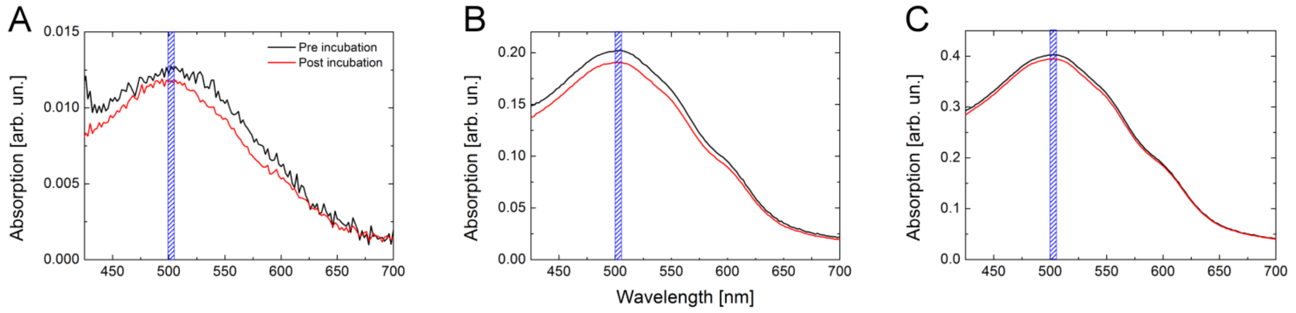

Fig. S4. Variation of the optical absorption spectrum before and after 24 hours administration to cell cultures (black and red solid lines, respectively) at three different initial optical densities. (A) 0.05; (B) 0.2; (C) 0.4. Absorption spectra have been obtained as the average over n = 6 independent samples. The blue rectangle indicates the spectral region over which the relative differences of the optical absorption have been evaluated.

Characterization of PS NPs

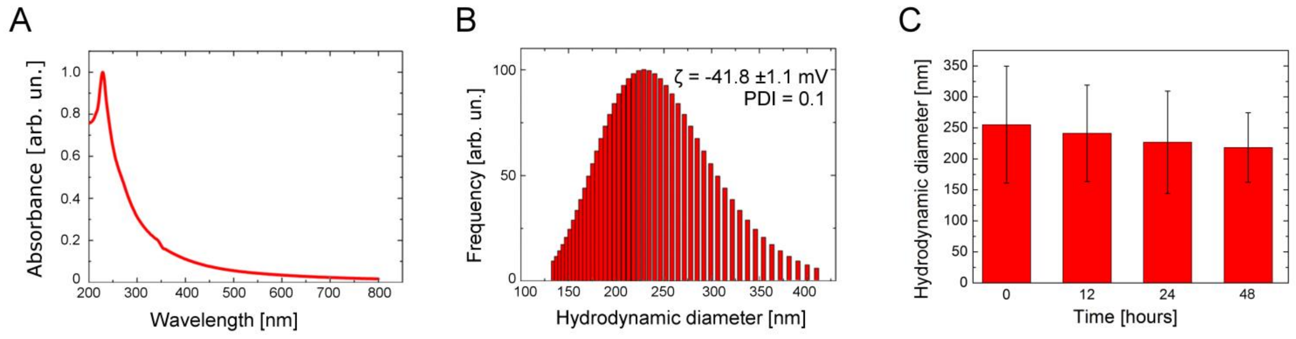

Fig. S5. (A) Electrically inert NPs, used as control samples, are entirely composed of polystyrene (PS NPs) and do have negligible absorption in the visible range. (B) PS NPs dimensions have been evaluated by DLS measurements, obtaining average values of 225 ± 72 nm, fully comparable to the ones of P3HT NPs. The zeta-potential is negative, with values amounting at -41.8 ± 1.1 mV. (C) Temporal stability tests in D-MEM cell culturing medium. No statistically significant changes in the hydrodynamic diameter values are observed over time.

Chronoamperometry measurements on different polymer substrates

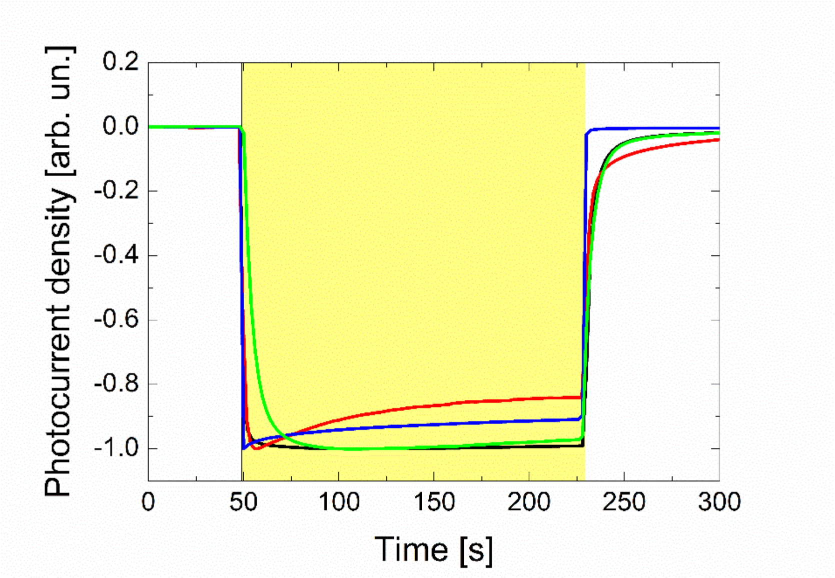

Fig. S6. Comparison among chronoamperometry signals obtained in P3HT NPs dispersion (red solid line), P3HT thin films spin-coated from solution onto the ITO slab (blue solid line), P3HT NPs drop-casted from dispersion (black solid line) and ITO electrode previously exposed to NPs dispersion (green solid line).

Ca^{2+} dynamics data set

Ca^{2+}-rich extracellular medium

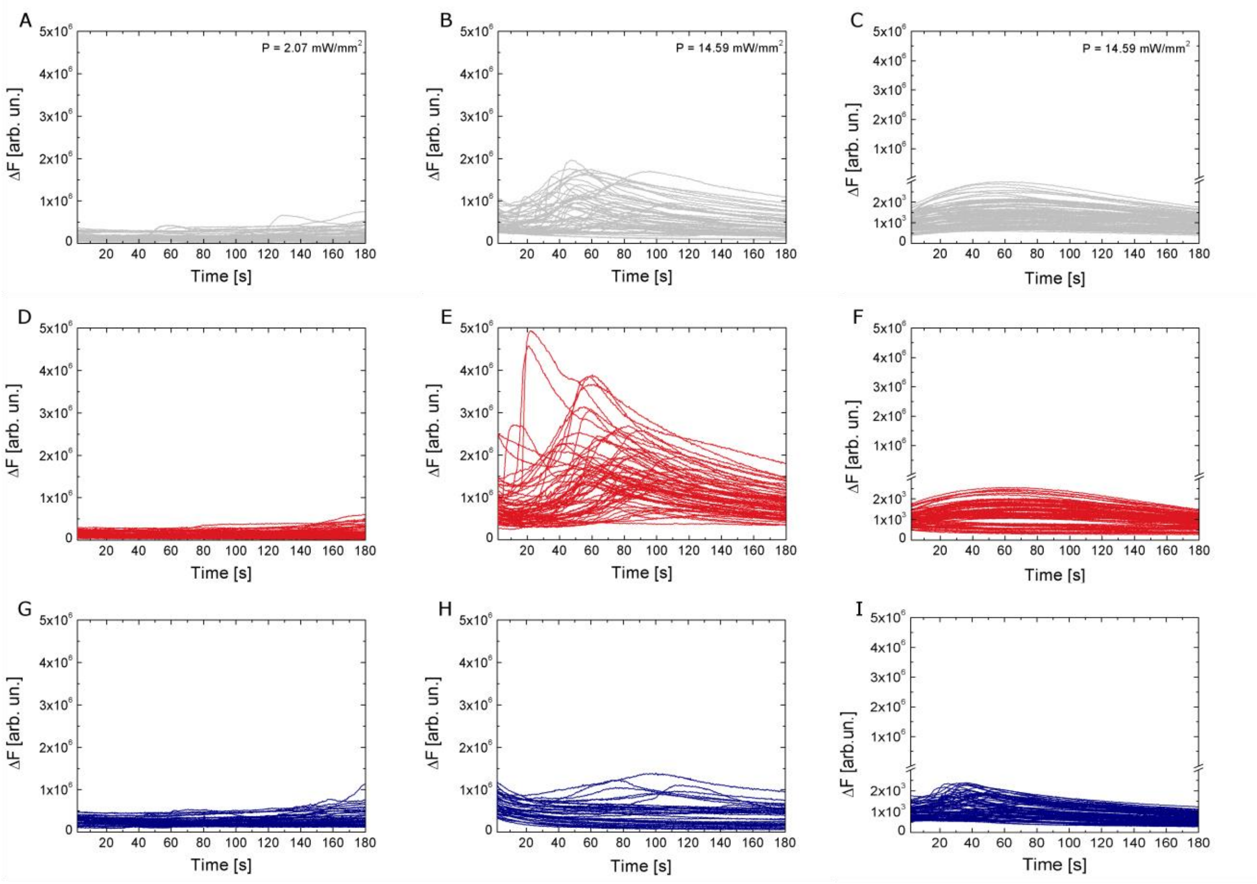

Fig. S7. Calcium dynamics recorded in HEK-293 cells cultured on control glass substrates (grey lines), treated with P3HT NPs (red lines) and treated with light-insensitive PS NPs (blue lines). Left panels (A, D, G): data recorded at excitation density of 2.1 mW/mm^2. Middle panels (B, E, H): data recorded at excitation density of 14.6 mW/mm^2. Right panels (C, F, I): data recorded at excitation density of 14.6 mW/mm^2, upon NAC administration.

Ca^{2+}-free extracellular medium

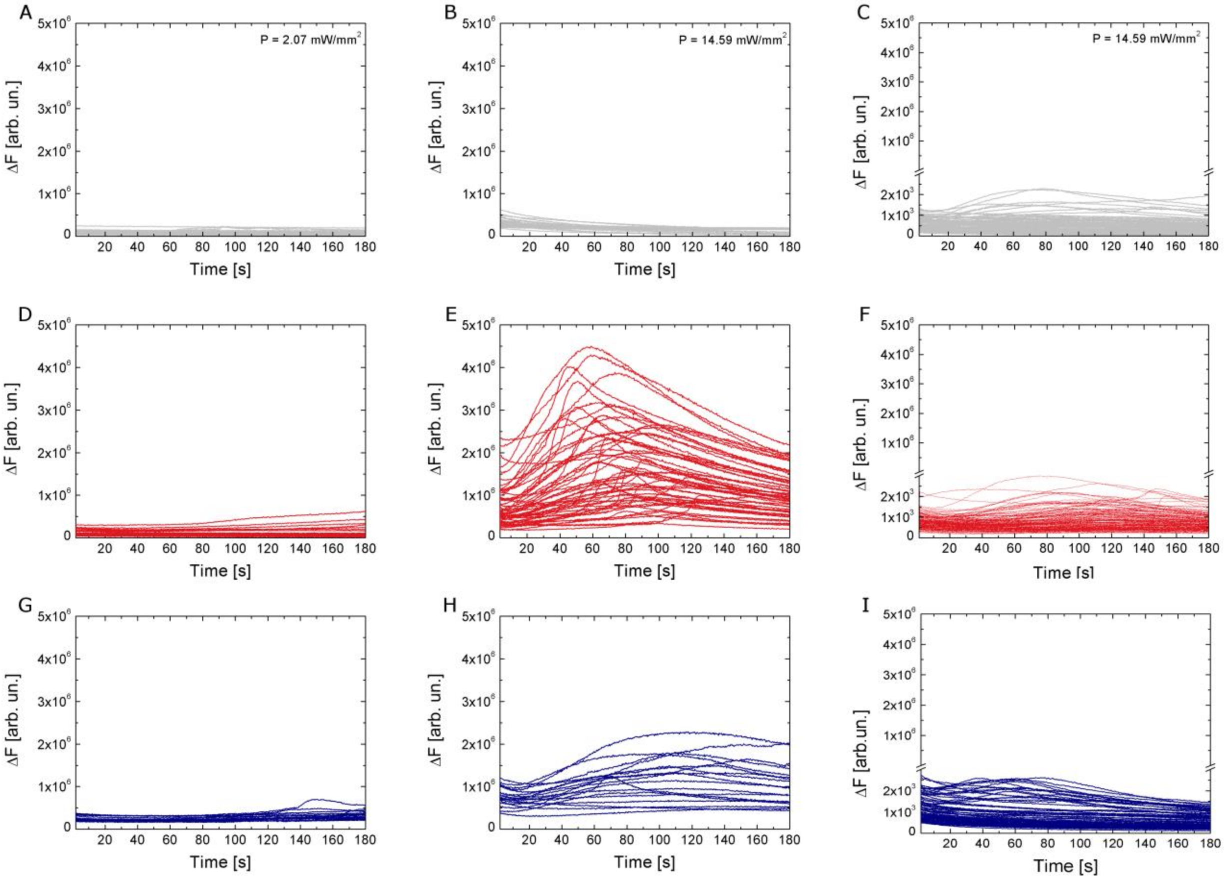

Fig. S8. Calcium dynamics recorded in HEK-293 cells cultured on control glass substrates (grey lines), treated with P3HT NPs (red lines) and treated with light-insensitive PS NPs (blue lines), in absence of extracellular Ca^{2+}. Left panels (A, D, G): data recorded at excitation density of 2.1 mW/mm^2. Middle panels (B, E, H): data recorded at excitation density of 14.6 mW/mm^2. Right panels (C, F, I): data recorded at excitation density of 14.6 mW/mm^2, upon NAC administration.

Measurements on HEK-293 cells transfected with TRPV1 channel, treated with P3HT NPs

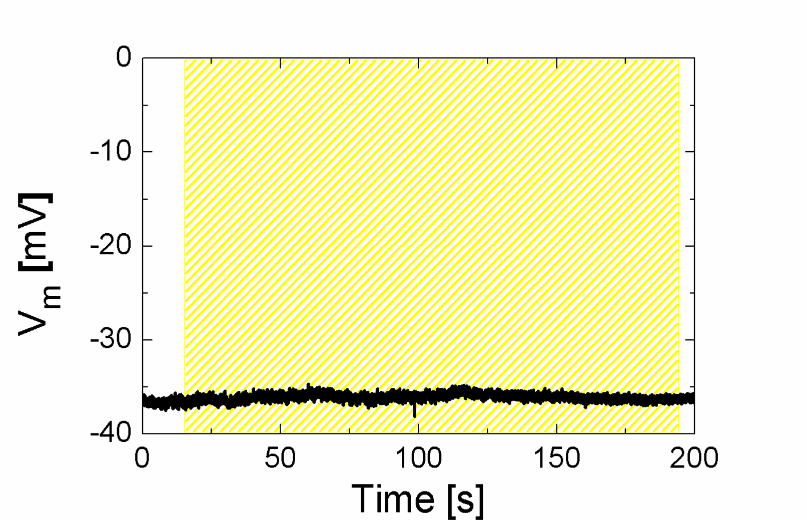

Fig. S9. Patch clamp experiments on HEK-293 cells expressing temperature sensitive TRPV1 channels, in current clamp configuration (I = 0). Light stimulus is represented as a yellow rectangle. No significant variation is recorded upon light in the resting membrane potential V_m.

Effect of the light stimulation protocol on cell viability

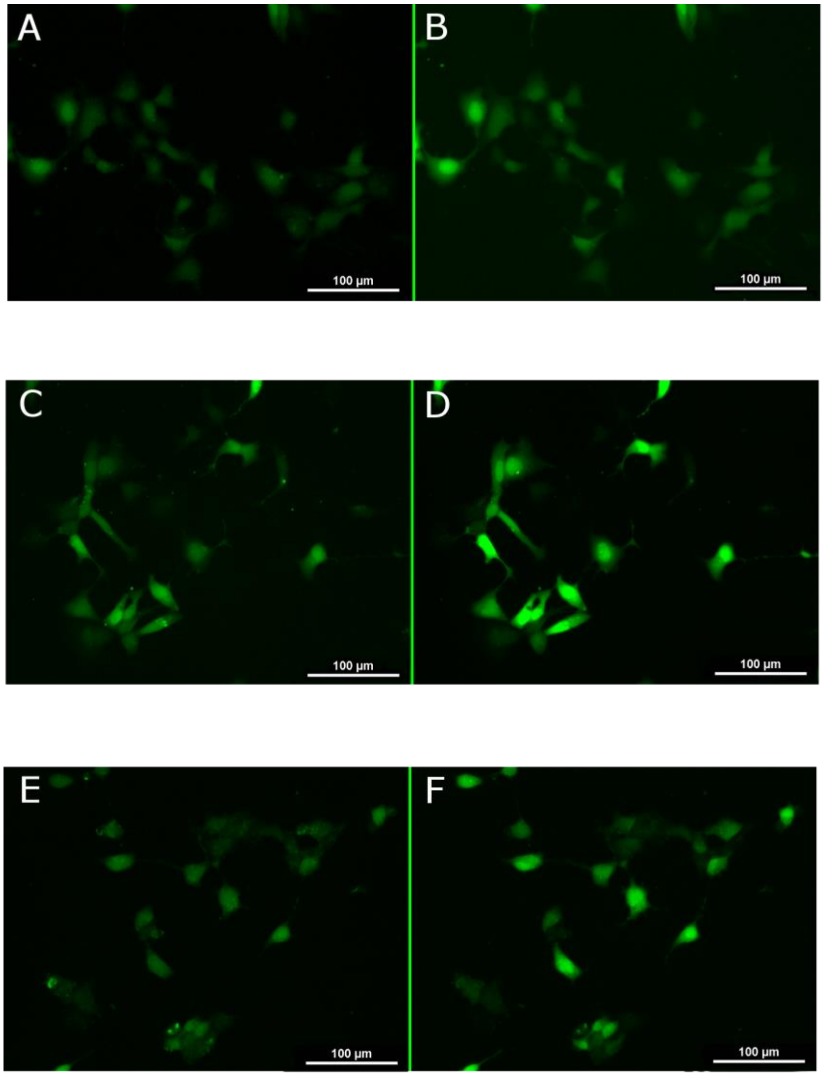

Fig. S10. Fluorescence images of HEK-293 cells cultured on control glass substrates (A, B), treated with P3HT NPs (C, D) and treated with PS NPs (E, F). Left and right images are acquired before and after the photostimulation protocol, used for inducing production of ROS. In all cases, the cell morphology and viability are not significantly affected by the light excitation.

Statistical analysis

Data reported in Fig. 3, Fig. 4 and Fig. 5 have been analyzed by using the ANOVA test (one way, 0.01 significance level). Post-hoc analysis has been carried out by the Tukey test; p-values corresponding to all considered samples cohorts are reported in the following Tables 1- 8. Statistically significant differences have been observed in ROS production, evaluated with different probes, between samples treated with P3HT NPs and exposed to light, as compared to non-treated samples, samples treated with inert PS NPs and samples not exposed to light (Table 1). Similar conclusions have been obtained for evaluation of intracellular Ca^{2+} increase (Table 2). Statistical analysis has also been carried out on measurements at different P3HT NPs doses, both on ROS production (Table 3) and on Ca^{2+} dynamics modulation (Table 4). We observe a statistically significant difference in illuminated samples treated with increasingly higher NPs concentration, corresponding to initial optical densities of 0.2 and 0.4, with respect to control samples. Conversely, treatment with P3HT NPs at initial optical density of 0.05 does not show statistically relevant differences as compared to control samples.

	**H_2DCF-DA**				
		Untreated Light	Untreated Dark	PS NPs Light	PS NPs Dark
**p-value = 0**	P3HT NPs Light	8.7×10^{-9}	0	0.0005	0
	P3HT NPs Dark	0	0.02	0	0.002
	**APF**				
		Untreated Light	Untreated Dark	PS NPs Light	PS NPs Dark
**p-value = 0**	P3HT NPs Light	7.1×10^{-10}	0	0	0
	P3HT NPs Dark	0.58	0.07	0.03	0.65
	**HPF**				
		Untreated Light	Untreated Dark	PS NPs Light	PS NPs Dark
**p-value = 4.5×10^{-8}**	P3HT NPs Light	0	4.3×10^{-8}	4.5×10^{-8}	2.2×10^{-9}
	P3HT NPs Dark	0.04	0.97	0.14	0.08

Table 1. p-values obtained by the Tukey test applied to data of Figure 3A-3C (production of ROS evaluated by H_2DCF-DA, APF and HPF probes, respectively).

	Untreated	PS NPs
**P3HT NPs / Ca^{2+} rich**	5.4×10^{-5}	3.5×10^{-4}
**P3HT NPs /Ca^{2+} free**	1.6×10^{-8}	4×10^{-4}

Table 2. p-values obtained by the Tukey test applied to data of Figure 4B and 4D (variation of intracellular Ca^{2+} dynamics with complete extracellular medium and Ca^{2+} free extracellular medium).

	**P3HT NPs - OD 0.05**		**P3HT NPs - OD 0.2**		**P3HT NPs - OD 0.4**	
**H_2DCF-DA**						
	p-value = 0.06		p-value = 0.005		p-value = 1.2×10^{-8}	
	Light	Dark	Light	Dark	Light	Dark
Untreated Light	0.54	0.99	0.005	1	3.2×10^{-8}	0.99
Untreated Dark	0.56	0.99	0.004	1	2.9×10^{-8}	0.99
**HPF**						
	p-value = 1		p-value = 1.2×10^{-7}		p-value = 0	
	Light	Dark	Light	Dark	Light	Dark
Untreated Light	0.91	0.95	2.4×10^{-6}	0.99	0	0.006
Untreated Dark	0.34	0.52	5.2×10^{-7}	0.98	0	6.7×10^{-4}
**APF**						
	p-value = 0.11		p-value = 0		p-value = 0	
	Light	Dark	Light	Dark	Light	Dark
Untreated Light	0.1	0.02	0	0.79	0	0.02
Untreated Dark	0.56	0.15	0	0.12	0	0.99

Table 3. p-values obtained by the Tukey test applied to data of Figure 5A-5C (production of ROS for different concentrations of internalized P3HT NPs, evaluated by H_2DCF-DA, HPF and APF probes, respectively).

	OD 0.05 2.1 mW/mm^2	OD 0.2 2.1 mW/mm^2	OD 0.4 2.1 mW/mm^2	Untreated 14.6 mW/mm^2
OD 0.05 14.6 mW/mm^2	0.001			0.99
OD 0.2 14.6 mW/mm^2		2.2×10^{-8}		0.001
OD 0.4 14.6 mW/mm^2			0	0.0003

Table 4. p-values obtained by the Tukey test applied to data of Figure 5F (variation of intracellular Ca^{2+} dynamics at different doses of P3HT NPs and two photoexcitation densities, 2.1 mW/mm^2 and 14.6 mW/mm^2). Comparison between treated and untreated cells, subjected to photoexcitation density of 14.6 mW/mm^2, is also shown.

TEM analysis of HEK-293 cells incubated with P3HT NPs

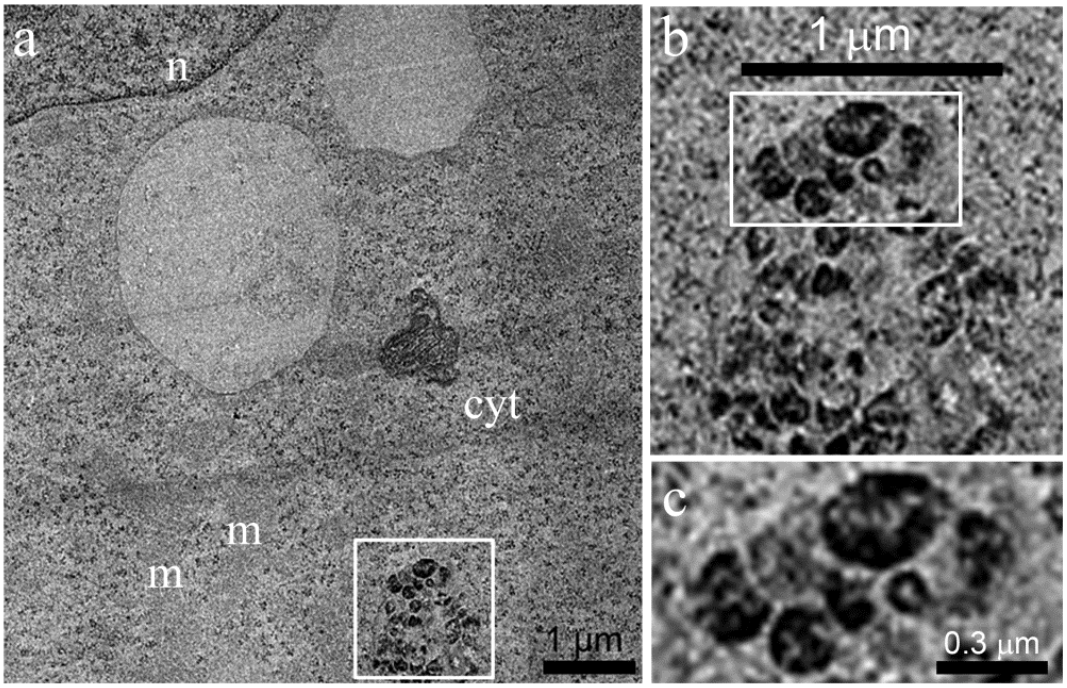

Fig. S11. TEM analysis of HEK293 cells incubated with P3HT NPs. (a) Representative transversal cell section, showing an endosome-like vesicle containing electron-dense nanoparticles (boxed region). (b) and (c) are higher magnification images of the boxed region in (a) and (b), respectively. Abbreviations: cyt, cytoplasm; m, mitochondria; n, nucleus.

Ca^{2+} dynamics at fixed time points.

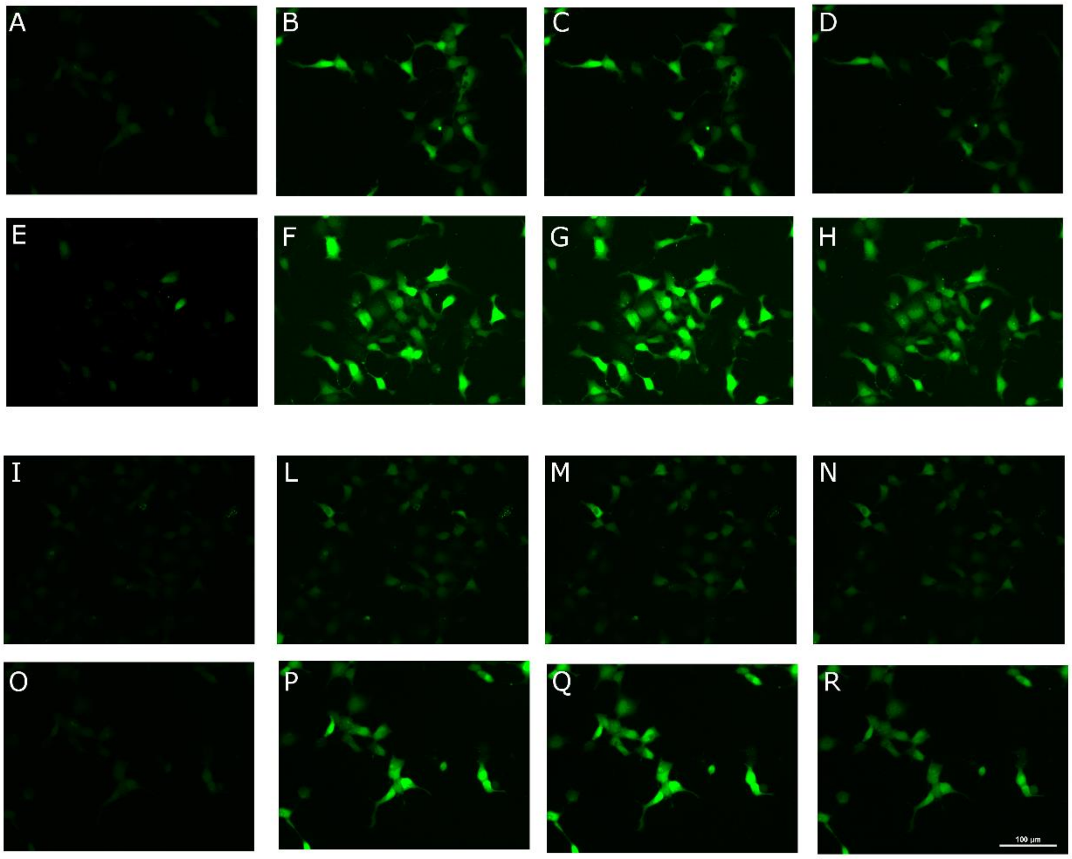

Fig. S12. Representative Fluo-4 fluorescence at fixed time points (0 s, 30 s, 100 s, 180 s), extracted from Ca^{2+} dynamics' measurements. (A-D) Untreated cells, complete KRH as extracellular solution. (E-H) P3HT NPs treated cells, complete KRH as extracellular solution. (I-N) Untreated cells, KRH Ca^{2+}-free extracellular solution. (O-R) P3HT NPs treated cells, KRH Ca^{2+}-free extracellular solution.
